# *Streptomyces coelicolor* macrodomain hydrolase SCO6735 cleaves thymidine-linked ADP-ribosylation of DNA

**DOI:** 10.1016/j.csbj.2022.08.002

**Published:** 2022-08-08

**Authors:** Andrea Hloušek-Kasun, Petra Mikolčević, Johannes Gregor Matthias Rack, Callum Tromans-Coia, Marion Schuller, Gytis Jankevicius, Marija Matković, Branimir Bertoša, Ivan Ahel, Andreja Mikoč

**Affiliations:** a Division of Molecular Biology, Ruđer Bošković Institute, Zagreb, Croatia; b Sir William Dunn School of Pathology, University of Oxford, Oxford, UK; c Division of Organic Chemistry and Biochemistry, Ruđer Bošković Institute, Zagreb, Croatia; d Department of Chemistry, Faculty of Science, University of Zagreb, Zagreb, Croatia

**Keywords:** ADP-ribosylation, DNA ADP-ribosylation, Macrodomain, Streptomyces, SCO6735

## Abstract

ADP-ribosylation is an ancient, highly conserved, and reversible covalent modification critical for a variety of endogenous processes in both prokaryotes and eukaryotes. ADP-ribosylation targets proteins, nucleic acids, and small molecules (including antibiotics). ADP-ribosylation signalling involves enzymes that add ADP-ribose to the target molecule, the (ADP-ribosyl)transferases; and those that remove it, the (ADP-ribosyl)hydrolases. Recently, the toxin/antitoxin pair DarT/DarG composed of a DNA ADP-ribosylating toxin, DarT, and (ADP-ribosyl)hydrolase antitoxin, DarG, was described. DarT modifies thymidine in single-stranded DNA in a sequence-specific manner while DarG reverses this modification, thereby rescuing cells from DarT toxicity. We studied the DarG homologue SCO6735 which is highly conserved in all *Streptomyces* species and known to be associated with antibiotic production in the bacterium *S. coelicolor*. SCO6735 shares a high structural similarity with the bacterial DarG and human TARG1. Like DarG and TARG1, SCO6735 can also readily reverse thymidine-linked ADP-ribosylation catalysed by DarT *in vitro* and in cells. SCO6735 active site analysis including molecular dynamic simulations of its complex with ADP-ribosylated thymidine suggests a novel catalytic mechanism of DNA-(ADP-ribose) hydrolysis. Moreover, a comparison of SCO6735 structure with ALC1-like homologues revealed an evolutionarily conserved feature characteristic for this subclass of macrodomain hydrolases.

## Graphical abstract

**Figure:**
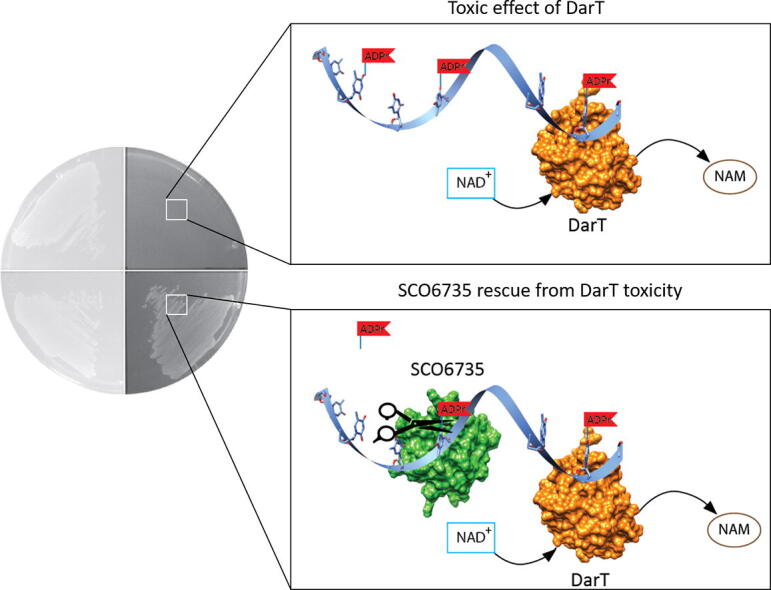

## 1 Introduction

ADP-ribosylation is an evolutionarily conserved, reversible chemical modification utilized by all domains of life and viruses [1], [2], [3], [4]. (ADP-ribosyl)transferases (ARTs) covalently attach adenosine diphosphate ribose (ADPr) from the metabolic cofactor NAD^+^ to different substrates, such as proteins, nucleic acids, and small molecules (including antibiotics), [5], [6], [7], [8], [9], [10]. The modification is reversed by two canonical but evolutionary distinct families - macrodomains and (ADP-ribosyl)hydrolases [5], [11]. In addition, there are other enzymes, like the phosphodiesterase NUDT16 and ENPP1, that can cleave the phosphodiester bond in ADPr, thus leaving the ribose-5′-phosphate attached to the protein [12], [13]. The macrodomain family shares a canonical α/β/α fold which consists of a six-stranded mixed β-sheet surrounded by five α-helices. The group can be further subdivided into six classes, including the three with hydrolytic activity: poly-(ADP-ribose) glycohydrolase PARG-like, MacroD-type and TARG1/ALC1-like macrodomains [14].

In eukaryotes, ADP-ribosylation has been implicated in a plethora of important cellular processes such as DNA repair, transcription, metabolism, apoptosis and pathologies such as cancer [4], [7], [15]. Although less characterized, ADP-ribosylation in bacteria also controls important pathways such as DNA-damage response [16], morphological differentiation and antibiotic production [17], [18], [19], [20]. The *Streptomyces* species are widely pharmaceutically exploited because of their ability to produce antibiotics and a wide range of pharmaco-active substances. Within their large genomes, *Streptomyces* encode for a wide range of ADP-ribosylation signalling enzymes catalysing protein, DNA and antibiotic modifications [5], [17], [18], [21], [22], [23], [24], [25], [26]. There are three characterized (ADP-ribosyl)transferases in *S. coelicolor* – SCO2860, SCO3953 and SCO5461. The SCO2860 is an (ADP-ribosyl)transferase that modifies antibiotic rifampin [27]. SCO3953 is a tRNA 2′-phosphotransferase that modifies 5′-phosphorylated RNA and is evolutionarily conserved from bacteria to humans [28]. SCO5461 is a pierisin homologue and has a guanine-specific DNA (ADP-ribosyl)transferase activity [29]. There are far more hydrolases in *S. coelicolor* found so far; eight of them are uncharacterized DraG/ARH homologues (SCO0086, SCO1766, SCO2028, SCO2029, SCO2030, SCO2031, SCO4435, SCO5809) and three are macrodomain hydrolases – SCO0909 (bacterial-type PARG), SCO6450 (MacroD homologue) and SCO6735 (ALC1-like). Both SCO6450 and SCO6735 can remove mono-ADP-ribosylation from protein substrates [30], [17]. Furthermore, SCO6450 can remove mono-ADP-ribosylation from phosphorylated 5′-RNA and both DNA ends [28], [30].

Although ADP-ribosylation was historically considered a protein modification, mounting evidence highlights its conserved roles in the modification of nucleic acids [6], [28], [31], [32], [33], [34], [35], [36], [37]. ADP-ribosylation of nucleic acids was first detected more than 20 years ago with the discovery of the pierisin transferase in cabbage butterfly which can ADP-ribosylate guanidine bases in dsDNA, [38], [39]. Its orthologues are also found in molluscs [36] and bacteria [40], including the homologue SCO5461 from *S. coelicolor*. It is the only studied endogenous *S. coelicolor* ART known to modify guanidines in any given DNA oligonucleotide sequence or guanidine-derived nucleoside [29]. Human PARPs have been shown to modify nucleic acids *in vitro*: PARP1-3 ADP-ribosylate the phosphate groups found at either DNA end [33], [34], [37], [41], TRPT1 (also known as PARP18) modifies 5′-phosphates on DNA and RNA ends [28], [42], and PARP10, PARP11 and PARP15 have been shown to ADP-ribosylate the 5′-phosphates at RNA ends [28]. The (ADP-ribosyl)hydrolases PARG, TARG1, MacroD1, MacroD2 and ARH3 can reverse phosphate-linked DNA and RNA ADP-ribosylation [28], [34].

Recently, the first reversible DNA-specific toxin/antitoxin (TA) pair DarT/DarG was discovered in several pathogenic bacteria (including enteropathogenic Escherichia coli (EPEC), Mycobacterium tuberculosis, Klebsiella pneumoniae and Pseudomonas mendocina) [32], [35], [43], [44]. DarT is a DNA-specific ART that modifies the second thymidine in the TNT(C) motif [31], [32], [35]. Thymidine ADP-ribosylation is sensed as DNA damage which causes impairment of bacterial growth and activation of the SOS response. The formation of the DNA-ADPr adduct is reversed via the action of the DarG macrodomain hydrolase [35], which is an essential gene when DarT is present [31], [32], [35], [43], [45]. In addition to its ability to remove DarT-mediated DNA ADP-ribosylation, DarG also counteracts DarT activity by physically sequestering the toxin [32]. The main function of the DarT/DarG system is believed to be providing growth control in bacteria [31], [32], [35] and antiphage defence [46] by preventing bacterial or viral DNA replication. In *M. tuberculosis* the DarT/DarG pair have an endogenous function by regulating the cell growth by ADP-ribosylation of DNA at the origin of chromosome replication. In this scenario, DarG functions as a non-canonical DNA repair enzyme [31]. DarG shares more sequence similarity to human TARG1 than to any other human macrodomain protein [47]. Overexpression of DarT in human TARG1 knockout cell lines causes a DNA damage response due to replication fork progression arrest. The rescue experiment reintroducing TARG1 activity shows that TARG1 is directly responsible for this reversal of DarT genotoxic effects. This suggests that TARG1 is the main macrodomain enzyme in human cells that acts as a DNA repair factor analogously to DarG [47]. TARG1 can cleave the acetal O-glycosidic bond present in nucleic acid substrates mono‐ADP-ribosylated at the phosphate ends [34], the acetal O-glycosidic bonds between the protein proximal ADPr and sidechains of acidic amino acids (Glu and Asp); and the acetate group in *O*-acetyl-ADPr (OAADPr) [48], [49].

Previous studies have suggested that the *S. coelicolor* hydrolase SCO6735 belongs to the same macrodomain subgroup as TARG1 and DarG [17], [50] and is the only such protein found in *S. coelicolor* [21]. SCO6735 was shown to reverse the protein ADP-ribosylation on glutamate residues, although the endogenous transferase is not known. SCO6735 gene is regulated by the highly conserved RecA-NDp-type promoter element that precedes numerous genes involved in DNA damage response in *Actinobacteria* [51], [52]. The deletion of SCO6735 leads to a “blue phenotype” due to the increased production of the antibiotic actinorhodin, which is indicative of its involvement in the cellular stress response. Concurringly, UV-irradiation increases SCO6735 expression. However, SCO6735 deficiency does not significantly influence survival rates after UV or MMS exposure. A comparison of the sequence and structure of SCO6735 with known homologues shows the absence of residues formally identified as crucial for catalysis, thus suggesting a mechanism diverged from the known macrodomains of this class [17].

Here, we show that SCO6735 represents a subclass of bacterial ALC1-like macrodomains which have an additional structural element enabling high activity against ADP-ribosylated thymidines modified by DarT. Using biochemical analysis and molecular dynamics simulation, we provide novel insights into the SCO6735 catalytic mechanism underlying thymidine de-modification.

## 2 Results

### 2.1 Thymidine ADP-ribosylation reversal: A unifying function of the ALC1-like macrodomain class

**Fig. 1:**
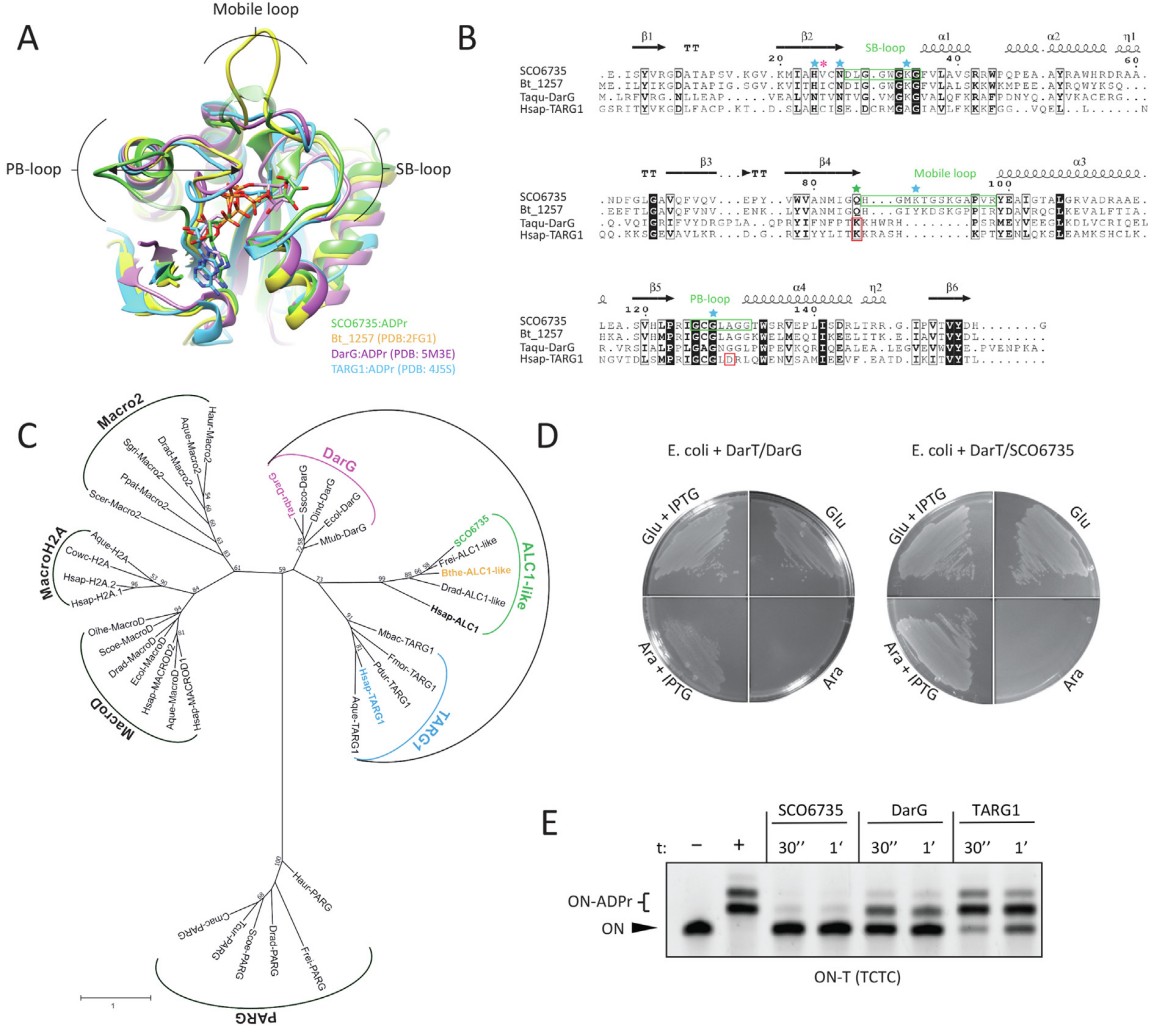
SCO6735 is a functional homologue of DarG and TARG1. (A) Superimposition of SCO6735:ADPr complex (green) obtained by molecular docking and four cycles of energy minimisation (for molecular docking we used crystal structure of SCO6735 (PDB 5E3B)) with Bt_1257 (yellow; PDB 2FG1), DarG:ADPr (pink; PDB 5M3E) and TARG1:ADPr (blue; PDB 4J5S). ADPr is depicted as a stick model. Different positioning of PB-loop is shown by an arrow. Loops missing in the SCO6735 crystal structure (PDB 5E3B) are built with the SWISS-MODEL and shown in transparent green. (B) Multiple sequence alignment of SCO6735, Bt_1257, TARG1 and DarG based on structural superimposition of all four proteins. Secondary structures are designated above sequences. Catalytically important amino acids in TARG1 and DarG are highlighted with red boxes. Gln85 in SCO6735, equivalent to catalytically important lysine in TARG1 and DarG, is highlighted with a green star. Residues marked with a blue star were analysed by mutagenesis studies, and Val25 (marked with a pink asterisk) we deemed important for the catalytic mechanism. (C) Phylogenetic tree of the macrodomain superfamily. The species' full names and accession numbers of the 38 macrodomain sequences involved are listed in Supplementary Table 1. The colours are the same as in (A). (D) SCO6735 can rescue *E. coli* from the toxic effect of DarT. Toxicity assay monitoring the growth of *E. coli* BL21(DE3) with pBAD33 carrying DarT and pET28 carrying DarG or SCO6735. Plates were supplemented with glucose/arabinose for suppression/induction of expression from the pBAD vector, glucose and IPTG for induction of expression from the pET vector. (E) SCO6735, DarG and TARG1 (0.5 µM) activity on DarT-modified DNA oligonucleotide (0.5 µM). The reactions were stopped after 30 s or 1 min and analysed by the gel-shift assay.

BLAST search recovered SCO6735 homologues present in almost all *Streptomyces* and a vast number of *Actinobacteria* (data not shown). Dali search (https://ekhidna2.biocenter.helsinki.fi/dali/) [53] revealed structural homologues of SCO6735 (PDB 5E3B) within the Protein Data Bank. Structures of Bt_1257 protein from *Bacteroides thetaiotaomicron* (PDB 2FG1; Z-score of 25.5; RMSD 0.9 Å), DarG macrodomain from *Thermus aquaticus* (PDB 5M3E; Z-score of 17.1; RMSD 2.3 Å) and human TARG1 protein (PDB 4J5S; Z-score of 16.4; RMSD 2.2 Å) showed remarkable structural similarity to SCO6735 (Fig. 1A). Sequence alignment only partially reflected that score (SCO6735 shared 54 % with Bt_1257, 23 % with DarG and 17 % sequence identity with TARG1) (Fig. 1B). The superimposition of SCO6735 crystal structure with TARG1 and DarG in complex with ADPr revealed a putative active site confined by three loops (Fig. 1A and [17]). Loops that enclose the central part of the substrate-binding cleft are characteristic of all macrodomain proteins. The diphosphate and distal ribose are usually accommodated between two loops [14]. In SCO6735 we named these two loops the phosphate-binding (PB) and the substrate-binding (SB) loop (Fig. 1A). Although all four homologues have a third loop, it is five amino acids longer in SCO6735 and Bt_1257. Because of its dynamic properties observed during MD simulations, we named it the mobile loop (Fig. 1A and B). Alignment of SCO6735 with its homologues from different species (structures predicted with Alpha fold [84]) revealed that the longer mobile loop is not only conserved within the *Actinobacteria* and *Bacteroidetes* but is characteristic for this subclass of ALC1-like macrodomains (Supplementary Fig. 1).

Phylogenetic analysis shows that SCO6735, Bt_1257, TARG1 and DarG cluster within the ALC1-like class of macrodomains. Interestingly, we found further subdivisions in this class with ALC1-, DarG- and TARG1-like macrodomains forming distinct groups (Fig. 1C). To explore whether SCO6735 could be functionally related to DarG/TARG1, we tested if SCO6735 could reverse the toxic effects of DNA ADP-ribosylation on thymidines established by DarT in *E. coli*. To that end, we utilised a system in which the toxin DarT is co-expressed with potential *T*-ADPr hydrolases [35]. Under conditions that allow unimpeded DarT function, bacterial growth is arrested. However, if *T*-ADPr hydrolase activity is present, DarT activity is counteracted, and the bacterial growth is restored. As expected, DarG acted as an antitoxin to DarT. The same effect was observed when DarT and SCO6735 were co-expressed (Fig. 1D). This result shows that SCO6735 is a functional homologue of DarG.

Using the DarT-modified DNA oligonucleotide (termed ‘TCTC oligo’), we compared the efficiency of hydrolysis of SCO6735, DarG and TARG1 using a gel-shift assay (Fig. 1E). SCO6735 showed the highest efficiency as almost all of the substrate was hydrolysed after half a minute.

**Fig. 2:**
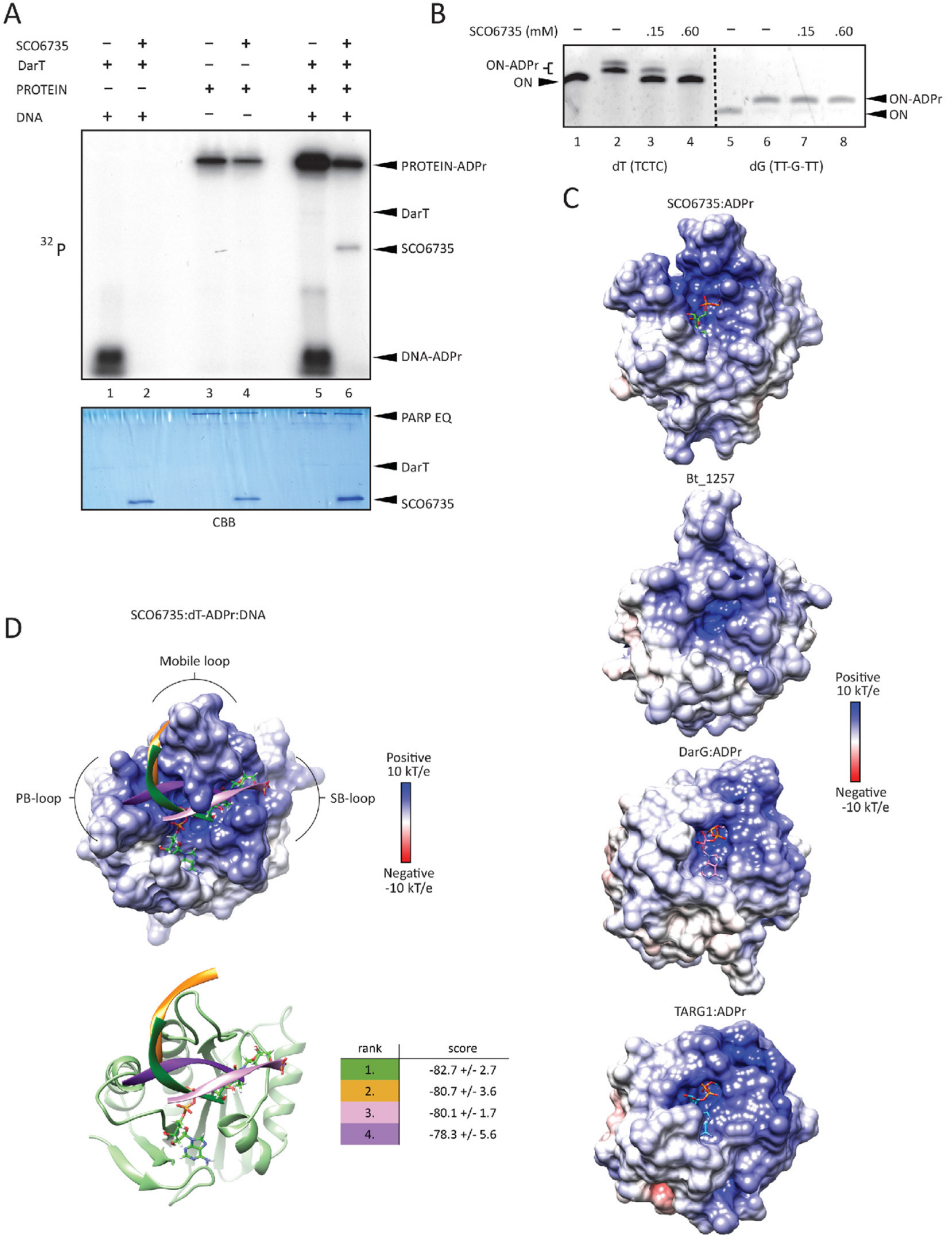
Binding and efficiency of SCO6735 on dT-ADPr DNA substrate. (A) SCO6735 activity (1 µM) on DNA-ADPr (1 µM, lanes 1 and 2), protein-ADPr (1 µM, lanes 3 and 4) and competition reaction with DNA- and protein-ADPr with SCO6735 (2 µM) (lanes 5 and 6) using ^32^P-NAD^+^ as an ADPr donor, on polyacrylamide urea gel, autoradiograph (upper panel). Coomassie Brilliant Blue (CBB) staining of the same gel showing protein loading (lower panel). (B) The activity of SCO6735 on DarT modified TCTC vs SCO5461 modified TT-G-TT oligonucleotide substrate, analysed by gel-shift assay on polyacrylamide urea gel. (C) Comparison of the electrostatic surface potential of SCO6735:ADPr complex after 50 ns of MD simulation with crystal structures of the apo structure of Bt_1257 (PDB 2FG1); and DarG (PDB 5M3E) and TARG1 (PDB 4J5S) in complex with ADPr. (D) Docking of DNA molecule in the open binding site of SCO6735. The best four docking scores are listed in the table. The colour of the docking score corresponds to the predicted binding mode of the DNA molecule in the figure. Bound dT-ADPr is depicted as green sticks.

Since SCO6735, as well as TARG1, de-modifies both DNA and protein [34], [47], [48], we compared its relative activity on both of these substrates (Fig. 2A). The DarT-modified TCTC oligo and the auto-ADP-ribosylated PARP1 E988Q mutant were used as substrates [48]. DNA ADP-ribosylation was thoroughly removed by SCO6735, while the protein ADP-ribosylation was not (Fig. 2A, lanes 2 and 4). This difference was more obvious in competition reaction where these substrates were used together (Fig. 2A, lanes 5 and 6). To compare its activity on protein to the one of TARG1, we tested the SCO6735 residue specificity on different protein substrates and confirmed that SCO6735, like TARG1 [48], hydrolyses the Glu/Asp-ADP-ribosylated proteins (Supplementary Fig. 2). Taken together, we conclude that SCO6735 shares the same substrates with TARG1.

Since SCO6735 has a higher activity against *T*-ADPr DNA than TARG1 and DarG, we decided to focus on understanding the catalysis of the de-ADP-ribosylation of the DNA-ADPr. To corroborate SCO6735 nucleotide specificity we used the TCTC oligo modified on thymidine (dT) and polyT-G (TT-G-TT motif) oligo modified on guanosine (dG), the products of DarT and SCO5461 transferases, respectively. SCO6735 efficiently de-modified dT-ADPr while having no activity against dG-ADPr (Fig. 2B). We, therefore, used dT-ADPr as the cognate substrate for studying the molecular mechanism of SCO6735 catalysis, while the dG-ADPr served as the negative control, *i.e.*, the non-cognate substrate.

### 2.2 Structural and dynamical properties of the SCO6735:substrate complex

The most obvious difference between the crystal structures of SCO6735 (PDB 5E3B, conformations used depicted in Supplementary Fig. 3A and B) and TARG1/DarG (PDB 4J5S/5M3E) is the positioning of the PB-loop. It was previously shown that in TARG1 and DarG, the PB-loop always adopts a closed conformation – both in apo form and in complex with the ADP-ribose [35], [48]. On the other hand, the PB-loop in the SCO6735 crystal structure in apo form is open and located far away from the active site (Fig. 1A and in [17]).

**Fig. 3:**
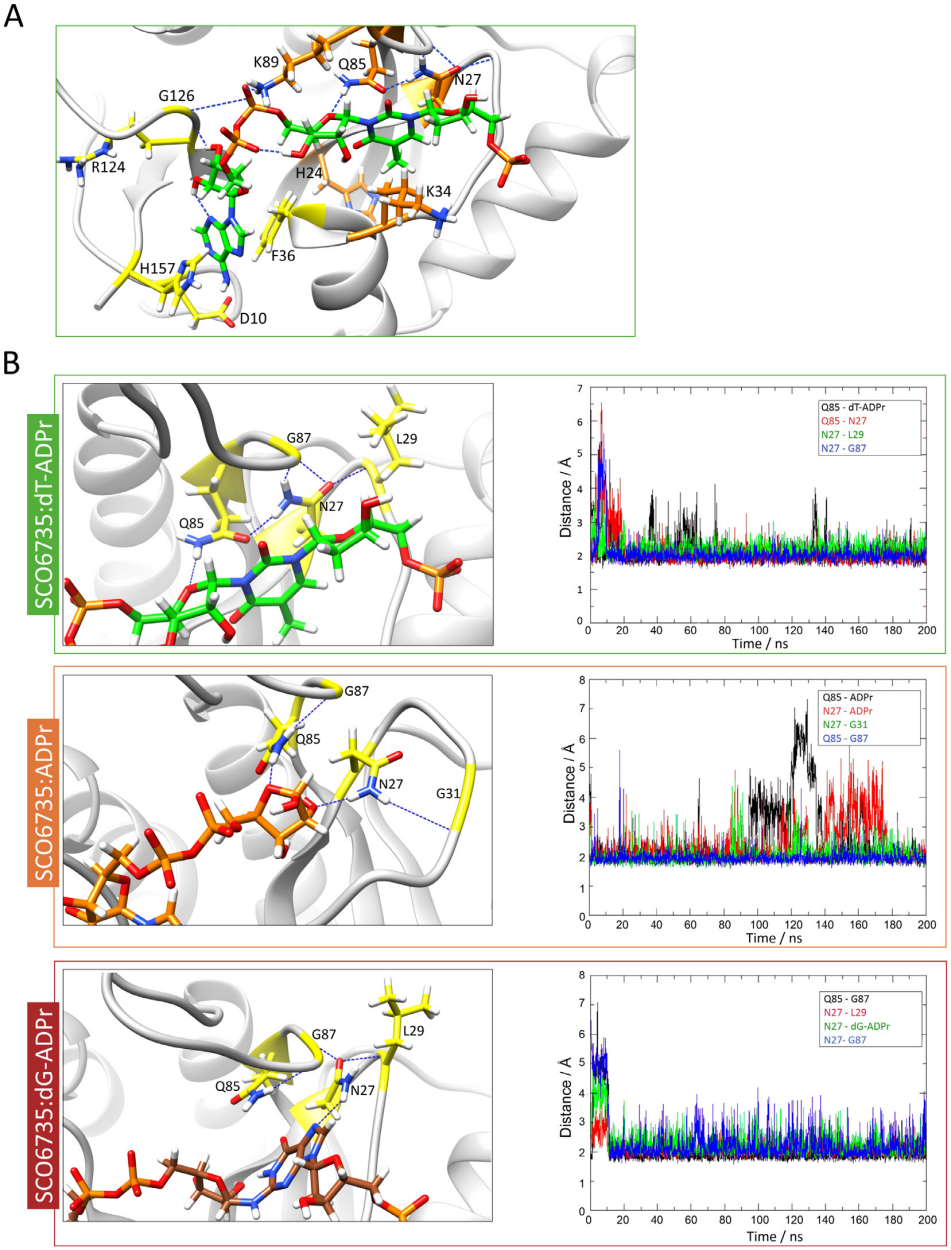
Gln85 in the SCO6735 active site has an important role in productive substrate binding. (A) A close-up view of the SCO6735:dT-ADPr complex active site obtained after 150 ns of MD simulation. Amino acids that make stable H-bonds or π-π stacking interactions during all MD simulations are depicted as sticks. Amino acids that were mutated are coloured orange. (B) Close-up view of main interactions with Gln85 in complex with the cognate substrate, product, and non-cognate substrate, respectively. The stability of labelled H-bonds is shown on the adjacent graph.

To get a better insight into the molecular dynamics of the SCO6735:substrate and SCO6735:product complexes, computational simulations were conducted. Complexes with the product (ADPr), cognate (dT-ADPr) and non-cognate (dG-ADPr) substrate were obtained via the molecular docking method (Supplementary Fig. 3D-F) and then subjected to molecular dynamics (MD) simulations. The atomic linkage of the ADPr to guanine bases was previously characterised using mass spectrometry [29], while the thymine-ADPr linkage was determined by NMR and crystallographic studies [31]. The 5′-phospho-deoxyribose was added to ADP-ribosylated bases to form a relatively small hydrophilic substrate that imitates the modified DNA molecule (2D schemes of cognate and non-cognate substrates are provided in Supplementary Fig. 3C).

During the MD simulation, the PB-loop moves towards and away from the active site, especially in the case of the apoprotein and the complex with the cognate substrate. Although some of the conformations sampled during the simulation with the cognate substrate were slightly closed, the PB-loop never adopted a fully closed conformation as in the crystal structures of DarG:ADPr (PDB 5M3E) and TARG1:ADPr (PDB 4J5S) complexes (Supplementary Fig. 4). Moreover, in the SCO6735:ADPr complex, the PB-loop did not move considerably (Supplementary Fig. 4). Comparing the MD simulations of the protein complexes with dT-ADPr and dG-ADPr revealed different positioning of an SB- and mobile-loop which are a consequence of different H-bond interactions between the Gln85, neighbouring amino acids and the ligand, modulating the active site conformation.

**Fig. 4:**
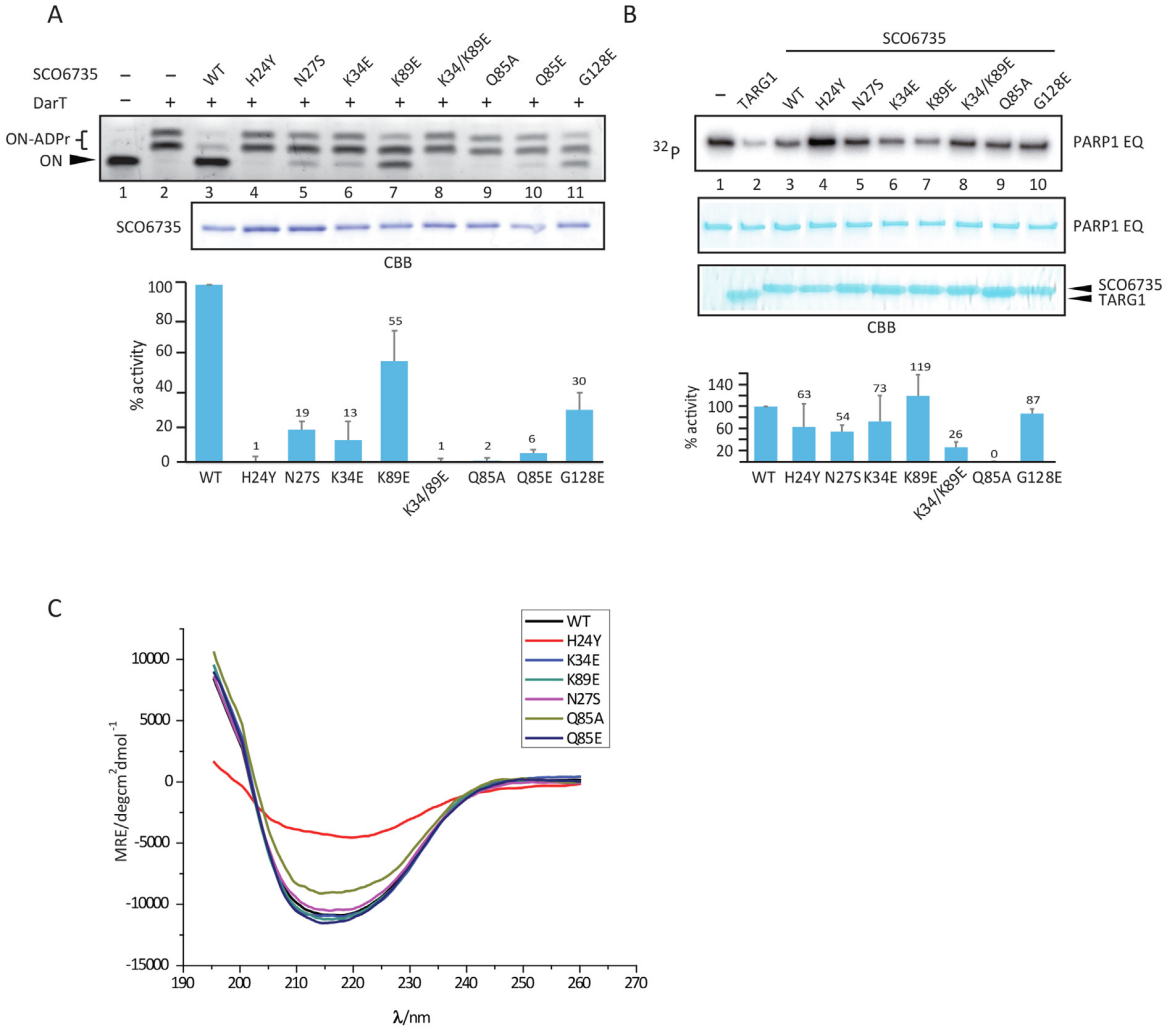
Contribution of SCO6735 active site residues to the activity on DNA and protein substrates. (A) SCO6735 WT and indicated mutants (0.1 µM) activity on DarT modified TCTC oligo analysed by gel-shift assay on polyacrylamide urea gel (upper panel); CBB staining of WT and SCO6735 mutants on SDS-PAGE gel used in the upper panel experiment, 30 µM of each protein loaded; quantifications of three independent experiments showing modified/non-modified oligo signal measured as pixel intensity, presented as the mutant/WT SCO6735, N = 3. (B) SCO6735 WT and indicated mutants (0.1 µM) activity on PARP1 E988Q mutant mono-ADP-ribosylated on Glu/Asp using ^32^P- NAD^+^ as an ADPr donor, autoradiograph (upper panel); CBB staining of the same gel as in the upper panel showing protein input; quantifications of ADPr-PARP1 E988Q signal in the autoradiograph normalized to the PARP1 EQ input and shown as mutant/WT SCO6735. Mean of activity written above each column, N = 3. Uncropped CBB and autoradiograph images can be found in Supplementary Fig. 9A. (C) Average CD isothermal spectra were calculated for SCO6735 WT, H24Y, K34E, K89E, N27S, Q85A and Q85E solutions (23 µM).

### 2.3 The electrostatic surface potential of the SCO6735 binding site is optimal for DNA-binding

A comparison of the electrostatic potential of the three enzymes' active sites could potentially explain the SCO6735 efficiency on DNA substrates and high turn-over rate compared to TARG1 and DarG (Fig. 1E and 2A). The surrounding area of all three active site clefts form an electropositive channel that is well suited for binding the single-stranded negatively charged DNA, and in the case of SCO6735 more electropositive residues are exposed (Fig. 2C). This pronounced electro-positivity arises from the longer mobile loop that contains two additional lysine residues (Lys89 and Lys93) not present in TARG1 and DarG (Fig. 1B).

Our hypothesis that in SCO6735 the single-stranded DNA could be enclosed by the mobile loop in the cleft formed between PB- and SB-loop is supported by the protein-DNA docking calculations carried out with the Haddock webserver [54], [55] (Fig. 2D). Four best-ranked DNA-binding modes in the SCO6735 active site were positioned in the positively charged groove surrounded by the PB-, SB- and mobile loop. We presume that a longer DNA strand could slide through the electropositive groove with the mobile loop acting as a clamp and ensuring a more effective DNA binding. This, together with the mentioned highly electropositive surface, would contribute to the greater processivity that we observed for SCO6735, compared to TARG1 and DarG (Fig. 1E, 2C and D).

### 2.4 SCO6735 active site revealed a novel catalytic mechanism within the ALC1-like macrodomain class

A comparison of the amino acids in the active sites of TARG1 and DarG to those in SCO6735 made it clear that the residues identified as catalytically important earlier in TARG1 (Lys84 and Asp125) [48] and DarG (Lys80) [35] are absent in the SCO6735 (Fig. 1B).

This led us to explore other amino acids in the active site using the interaction information obtained with MD simulations of SCO6735 in complex with the product and the cognate substrate. The potential roles of active site amino acids were determined using information about substrate and product-binding gained through MD simulations together with site-directed mutagenesis and *in vitro* gel-shift assays for activity testing.

In both, TARG1 and DarG, lysines in the active site are important for catalysis and while not isostructural, two lysines near the SCO6735 active site - Lys34 and Lys89 were observed to make important contacts with the substrate (Fig. 3A). Lys34 makes short-term H-bonds with the distal ribose and terminal phosphate (present through 8 % of the trajectory) and Lys89 establishes a stable interaction with the diphosphate (present through 52 % of the trajectory) (Supplementary Fig. 5A). Therefore, we decided to test their contribution to the SCO6735 enzymatic activity. Mutation of Lys34 to glutamate showed an almost complete loss in the enzyme activity on DNA (Fig. 4A, lane 6), while the K89E mutant showed a less prominent decrease of activity (Fig. 4A, lane 7). Nevertheless, when a K34E/K89E double mutant was used, we observed a complete loss of activity (Fig. 4A, lane 8). Based on the interactions observed during MD simulations and the fact that none of the single lysine mutations leads to the complete loss of activity, we concluded that they stabilize the negative charge of the DNA molecule and are important for its positioning.

**Fig. 5:**
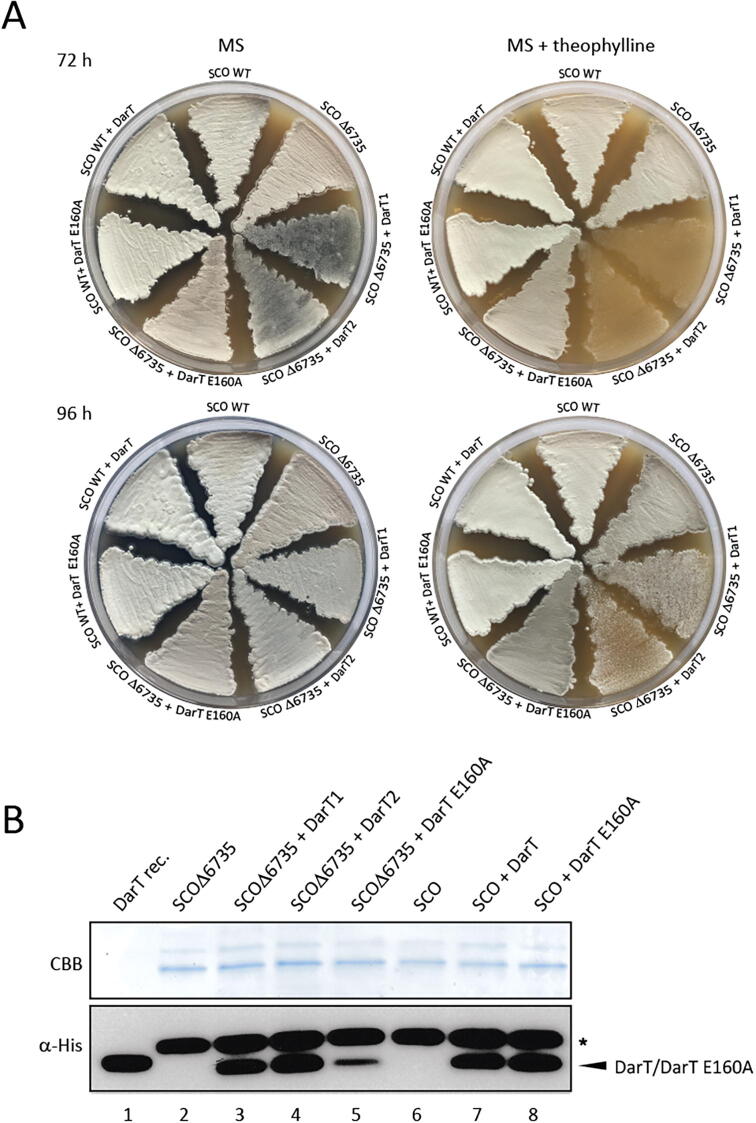
SCO6735 acts as an antitoxin of DarT in *S. coelicolor*. (A) *S. coelicolor* WT and SCO6735 deficient strain (SCOΔ6735) expressing DarT and DarT E160A catalytic mutant were grown on MS agar plates with (4 mM) or without theophylline. Growth was monitored for 96 h. (B) Western blot analysis of His-tag enriched total cellular proteins from the *S. coelicolor* strains expressing DarT and DarT E160A mutant. Two SDS-PAGE with identical samples were run in parallel; one was stained with CBB as a loading control (upper panel) and the other was used for detection of DarT/DarT E160A proteins using an anti-His antibody (lower panel). Purified recombinant DarT protein was used as the positive control (lane 1). Asterisk marks the background band seen in all cell lysate samples. Uncropped CBB and western blot images can be found in Supplementary Fig. 9B.

Mutation of Gln85, the equivalent to the main catalytic residues Lys84 in TARG1 and Lys80 in DarG [35], [48], into alanine led to a complete loss of enzyme activity (Fig. 4A, lane 9). MD simulation of SCO6735 in complex with cognate substrate showed that nitrogen from Gln85 amide group makes a very stable H-bond with the in-ring oxygen atom of the distal ribose (present through 62 % of the trajectory, Supplementary Fig. 5B). Further analysis of MD simulations of all three complexes (SCO6735 with ADPr, dT-ADPr and dG-ADPr) showed complex-specific differences in interactions between Gln85, the respective ligand and different neighbouring residues (Fig. 3B). For example, distal ribose of non-cognate substrate is positioned far away from the Gln85 (Supplementary Fig. 5B), which adopts a different rotamer (Fig. 3B). In complex with dT-ADPr Gln85 is carefully positioned by stable H-bonds with Gly87 and Asn27 which is further stabilised by Leu29. In this position, Gln85 can make a stable interaction with the in-ring oxygen atom of distal ribose (Fig. 3B). In the case of the dG-ADPr substrate, Gln85 is less buried in the active site and makes a stable H-bond with Gly87 which locks it in a different rotamer (Fig. 3B).

To further inspect the importance of stabilisation of Gln85 by Asn27 in complex with dT-ADPr, we decided to mutate Asn27 into serine (which would make it an equivalent of Ser26 in TARG1, Fig. 1B). This mutation was intended to remove the H-bond to Gln85 and make it more flexible. The SCO6735 N27S mutant showed an almost complete loss of activity (Fig. 4A, lane 5), while the overall protein structure was conserved (Fig. 4B, purple line). This result implies that Asn27 in SCO6735 is important for enzymatic activity most likely by locking Gln85 in the right rotamer which can interact and stabilise the cognate substrate. Interestingly, Asn27 (Fig. 1B) is conserved in DarG homologues and almost all enzymatically active macrodomains [56]. When mutated in DarG, it also led to a great loss in substrate turnover [35].

The Gly128 is conserved in all four homologues. Its mutation into glutamate (G128E) was shown to reduce SCO6735 catalytic activity [17]. In our gel-shift assay, we observed a substantial loss of activity (Fig. 4A, lane 11). Since the small Gly128 is positioned within the PB-loop it is probably important for the loop flexibility. The mutation into glutamate would lead to a more rigid structure of the PB-loop and adds a negative charge which repulses DNA in the active site.

Next, we tested the contribution of His24 to SCO6735 activity. His24 was positioned near the active site and was the only amino acid with the obvious catalytic potential. It was replaced with tyrosine to conserve the π-π stacking interactions established with the protein core and to remove the possibility of making H-bonds directed toward the substrate. We observed a complete loss of activity (Fig. 4A, lane 4), however, the circular dichroism spectrum of the mutant protein indicates that this loss of activity arises from a disrupted structure (Fig. 4C).

**Fig. 6:**
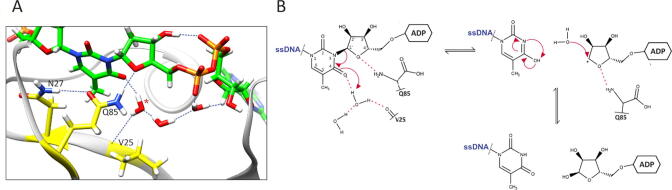
Proposed catalytic mechanism of SCO6735 protein on ADPr-dT-DNA substrate. (A) Val25 positions catalytic water molecule via the backbone oxygen atom. This water molecule (marked with the red asterisk), presumably, serves as a proton donor. (B) Proposed catalytic mechanism. The water molecule donates a proton to the second keto-oxygen atom in thymine which leads to the formation of the disfavoured tautomer, which immediately converses to the favoured form. The formed oxocarbenium ion presumably instantly reacts with a water molecule, resulting in ADPr as the reaction product. Gln85 seems to be crucial for the correct substrate positioning. Red dashed lines indicate H-bonds.

Since the mutation of Gln85 into glutamate or alanine showed complete loss of enzyme activity and it is known that glutamine is unable to partake directly in an acid-base reaction, we assumed that it could have an indirect role, such as a substrate or transition state positioning and/or stabilisation. The absence of the amino acid that could be directly involved in acid-base catalysis led us to the idea of a catalytic mechanism initiated by an activated water molecule. Having that in mind we performed a detailed analysis of the water molecules present in the active site during all MD simulations, which establish stable interactions with the cognate substrate near the catalytic centre. This revealed a water molecule nestled between Val25 and the second keto-oxygen atom (Fig. 6) in the thymidine ring during almost all of the MD simulation (Supplementary Fig. 6). This water molecule is also part of the water-molecule network between precisely positioned diphosphate and Gln85 (Supplementary Fig. 6A). Positioning of the diphosphate is further achieved through intramolecular H-bond with the proximal ribose (present through 60 % of the trajectory), Lys89 (51 % of the trajectory) and Gly126 (20 % of the trajectory) (Supplementary Fig. 5A).

To see whether there would be an overlap in the loss of enzyme activity on DNA-ADPr and protein substrates we tested the activity of SCO6735 mutants on auto-ADP-ribosylated PARP1 E988Q mutant. Most of the SCO6735 mutants lost about half of the activity (Fig. 4B). Single K34E mutant showed a rather small decrease in activity (Fig. 4B, lane 6), while the double mutant K34/K89E showed a strong decrease in activity on the protein substrate (Fig. 4B, lane 8). However, the Q85A was the only mutation with a consistent effect on the nucleic acid and the protein substrate and we observed the complete loss of activity on both substrates (lane 9 in Fig. 4A and 4B). These results suggest that SCO6735 employs a different reaction mechanism for de-ADP-ribosylation of protein substrates.

Since DarG inhibits DarT not only by hydrolysing its toxic product dT-ADPr but also by interaction-inhibition [32], [35], we wondered if SCO6735 also has this ability. Therefore, we used catalytically inactive mutants of SCO6735 (Q85A), DarG (K80A) and TARG1 (K84A) and measured DarT activity on DNA in their presence. Interestingly, we found that SCO6735 also efficiently inhibits DarT (Supplementary Fig. 7). Moreover, when compared to DarG and TARG1, SCO6735 showed the highest interaction-inhibition of the DarT toxin.

### 2.5 SCO6735 protects from DarT toxicity in *S. coelicolor*

To test if our results could be translated into the physiological context, we established a SCO6735-deficient *S. coelicolor* strain (SCOΔ6735), as well as WT and SCOΔ6735 strains conditionally expressing DarT and catalytically inactive DarT E160A under the theophylline riboswitch control (Fig. 5A). The presence of recombinant DarT and DarT E160A proteins in *S. coelicolor* strains was confirmed by western blot analysis using an anti-His antibody (Fig. 5B). The SCOΔ6735 strains harbouring the DarT WT (strain 1 and strain 2) showed growth retardation when the expression of DarT was induced. The presence of SCO6735 in the *S. coelicolor* WT strain prevented this effect. When the inactive form of DarT was expressed, no growth retardation was observed. Nevertheless, the growth of the SCOΔ6735 strains harbouring DarT WT caught up with the rest of the strains after 96 h (Fig. 5A). This means that DarT is not as toxic in *S. coelicolor* as it is in the *E. coli* and/or other endogenous mechanisms can metabolize the obstructive DNA-ADPr adducts. The same growth retardation effect was also observed when *S. coelicolor* strains were grown in liquid CRM medium (Supplementary Fig. 12).

## 3 Discussion

ADP-ribosylation is an evolutionarily and functionally conserved chemical modification mostly studied in the context of protein post-translational modification. For the past decade, though, there has been an increasing amount of evidence placing its origin in the modification of nucleic acids [5], [6], [10], [28], [31], [33], [34], [35], [41], [42], [57].

The DarT/DarG toxin/antitoxin pair is currently the best-characterised system for reversible DNA ADP-ribosylation [31], [35]. The lethal/toxic effect of DarT in the absence of DarG in bacteria was reported in *E. coli* and *Mycobacterium bovis*, indicating that in the absence of the corresponding repair enzyme, the lesions can induce cell death [31], [45]. Furthermore, when DarT was used as a genotoxin in human TARG1 knockout cells, it caused a severe DNA damage response at DNA replication sites. The effect was rescued only by constitutive expression of TARG1, reinforcing the idea that TARG1 and DarG share a similar function [47]. Here we show that their structural homologue SCO6735 from *S. coelicolor* can also neutralise the toxic effects of DarT in *E. coli* and *S. coelicolor*.

SCO6735 acts like DarG by efficiently and specifically de-modifying ADPr-*T*-DNA and neutralizing DarT by interaction-inhibition *in vitro*. When compared to TARG1 and DarG, SCO6735 most efficiently removed ADPr from modified DNA. Unlike DarG, SCO6735 also shows hydrolytic activity on proteins. Therefore, SCO6735 would encompass the functional range of the human TARG1. Structural comparison of DarG, TARG1 and SCO6735, showed high similarity of all three folds, yet hinted at a possible difference in the catalytic mechanisms. Previous studies of human TARG1 on ADP-ribosylated protein substrates led to the proposition of a catalytic mechanism carried out by a catalytic dyad involving Lys84 and Asp125 [48]. Deprotonation of Lys84 by Asp125 enables a nucleophilic attack of Lys84 nitrogen on the anomeric carbon atom of distal ribose, which leads to the liberation of the modified glutamate/aspartate. Subsequently, a Schiff base, susceptible to a nucleophilic water attack is formed. This leads to the formation of ring-opened ADPr and regeneration of the catalytic lysine [11].

DarG does not contain the catalytic Lys/Asp dyad, only the lysine residue remains conserved between the two enzymes [35]. Still, the complete catalytic mechanism remains elusive. As previously mentioned SCO6735 has neither of these catalytic residues, indicating a major mechanistic diversification within the ALC1-like class. Our studies revealed that mutation of Gln85 which is in an equivalent position as the catalytic lysine in DarG and TARG1 (Lys80 and Lys84, respectively) led to a complete loss of activity.

The fact that Glu85 or any of the residues in its structural vicinity cannot donate or subtract protons, and thus cannot be directly involved in an acid-base type reaction, led us to the assumption that the catalysis could be initiated by an activated catalytic water molecule. Indeed, a detailed analysis of the active site during MD simulation of the SCO6735:dT-ADPr complex revealed such water molecule nestled between Val25 and the second keto-oxygen atom in the thymine ring. Presumably, this water molecule could serve as a proton donor which would lead to the formation of the disfavoured thymidine tautomer as the reaction intermediate. Immediate tautomerization into the favoured form would hinder the backwards reaction, thus driving the reaction forward. Furthermore, the breakage of the dT-ADPr bond in this manner would generate a highly reactive oxocarbenium ion at the distal ribose. Given its solvent exposure and the absence of stabilising residues in the surrounding, we presume a short half-life of this intermediate, resulting in the formation of free ADP-ribose (Fig. 6B) [58].

This would make Val25 the main actor of the backbone-mediated catalytic mechanism in which it positions the water molecule via the backbone oxygen atom (Fig. 6). Since the MD simulation of a cognate substrate revealed a very stable H-bond between the Gln85 amide group and the in-ring oxygen atom of the distal ribose we presume that it is mainly responsible for substrate and transition state positioning (Fig. 3B and 6, Supplementary Fig. 6A).

The correct positioning of the Val25, Gln85 and the catalytic water molecule is ensured by the elaborate water-molecule network inside the active site that is positioned between these residues and the diphosphate of the ADP-ribose (Supplementary Fig. 6A).

The observed high processivity of SCO6735 on DNA substrate could be explained by the presence of the mobile loop which is five amino acids longer than in DarG/TARG1 (Fig. 1A and B) and contains two positively charged residues - Lys89 and Lys94 (missing in DarG/TARG1). Consequently, this would help DNA binding and stabilization of its negative charge. The mutation of Lys89 led to a considerable loss of activity on DNA but has an even better activity towards protein substrate. This differentiation of the binding site and relocation of the catalytic residues (Val25 and Gln85) to the opposite side of the binding cleft (compared to the other macrodomains) indicates possible adaptation to a dual role which enables de-modification of both DNA and protein substrates.

The SCO6735 homologues are widely present and highly conserved in Actinobacteria species. They can also be found in several other bacterial phyla (Bacteroidetes, Chloroflexi, Cyanobacteria, Deinococcus/Thermus, Firmicutes, Proteobacteria) (Supplementary Fig. 10). We found that even the cnidarian *Nematostella vectensis* has SCO6735 homologue as a part of a fusion protein which besides SCO6735 homologous macrodomain contains a domain from β-lactamase superfamily. This fusion is probably gained through horizontal gene transfer. It could indicate a connection between SCO6735 and antibiotic resistance since β-lactamases are a diverse class of enzymes produced by bacteria that can open the β-lactam ring, therefore inactivating the β-lactam antibiotics. The Val25 we deemed important for the catalytic mechanism of SCO6735 or chemically similar isoleucine in some cases, is a SCO6735 specific feature. The Gln85 important for the SCO6735 activity is conserved among both SCO6735 and ALC1 homologues and distinguishes them from the TARG1 and DarG homologues (Supplementary Fig. 10).

The effect of DarT in the SCO6735 deficient *S. coelicolor* strain was observed as a growth delay compared to the lethal effect in *E. coli*. This could be explained by fewer thymidine targets in *S. coelicolor* genomic DNA which has a GC content of 72 % and possibly has an additional repair mechanism in the *S. coelicolor,* besides SCO6735. It has been reported that DarG interacts with DNA repair factors such as RecA, RecB and RecF, the latter mediating the repair of ADPr-dT-DNA lesions [32], [45]. The same mechanism in *Streptomyces* could be combined with their slow growth and multicopy chromosome stage during sporulation [59]. This context would provide the opportunity for other DNA damage repair mechanisms to take place when DarT adducts, or their equivalent, are present in *S. coelicolor*. Since DarT homologues are present in more than a thousand bacterial species, we could assume that SCO6735 might act as direct protection from the other bacterial species bearing DarT-like excreted toxins. So far only one DNA-targeting ART has been found in *S. coelicolor* – SCO5461 (ScARP), a pierisin homologue that modifies N^2^ amino groups of guanine residues [29], [60]. It seems that SCO5461 is secreted toxin which *S. coelicolor* uses to fight nearby living bacteria.

BLAST search among *Streptomyces* species showed that some do harbour a DarT/G TA system, few of which have an additional SCO6735 gene and some, like the *S. coelicolor*, have only the SCO6735. We showed here that SCO6735 is a true functional homologue of DarG and therefore has the potential to be involved in anti-phage defence [46]. Although the fact that some *Streptomyces* species have both the SCO6735 and the DarT/G, implies an additional physiological role of SCO6735 aside from counteracting DarT. The upstream gene (SCO6734) has the same orientation as SCO6735 and encodes amino acid permease, an integral membrane protein involved in the transport of amino acids into the cell. In the intergenic region is a highly conserved RecA-NDp promoter which controls SCO6735 transcription, but still, we cannot completely rule out the possibility that these two genes can be transcribed together as a unique ORF in metabolic stress conditions (Supplementary Fig. 11).

Wide range of substrates (similar to TARG1), and the fact that its expression is under the RecA-NDp-type promotor point to a protective role of SCO6735 protein. This level of functional, even more than the structural, conservation from bacteria to humans might reflect the evolutionary pressure to keep this ALC1-like class of macrodomains as the protectors of the genome. Along the same line, the structural diversification we observed in SCO6735 longer mobile loop and the exchange of catalytic residues, could be the consequence of the same pressure to adapt and mechanistically diversify from the functionality of DarG towards that exhibited by the human TARG1.

## 4 Materials and methods

### 4.1 Plasmid constructs

Genes encoding DarT and DarG proteins from *Thermus aquaticus* together with their catalytic mutants were cloned as previously described in [35]; DarT and DarT E160A into pBAD33, DarG and DarG K80A into pET28a plasmid vectors.

The SCO6735 gene was PCR amplified from the *S. coelicolor* M145 genomic DNA and cloned into pET15b as previously described in [17]. For toxicity assay in *E. coli,* it was re-cloned into pET28b. Mutations were introduced using the asymmetric overlap extension PCR method [61] and mutated SCO6735 genes were also cloned into pET28b.

For the expression of DarT and DarT E160A in *S. coelicolor,* we used integrative plasmid pGusT-E* (a gift from Dr Michael-Paul Vockenhuber, Darmstadt University Technology, Germany) with the theophylline responsive synthetic riboswitch for the conditional gene expression [62].

All plasmid constructs were verified by sequencing.

### 4.2 Bacterial strains and culture conditions

TOP10 *E. coli* strain (Invitrogen) was used for all plasmid manipulations. DH5α/pBT340 strain (a gift from Dr Dušica Vujaklija, Ruđer Bošković Institute, Zagreb, Croatia) was used for the FLP recombinase-mediated excision of the disruption cassette central part, BL21 strain for the expression of DarT, BL21(DE3) for toxicity assay and the expression of SCO6735 mutants. The methylation-deficient *E. coli* strain ET12567/pUZ8002 was used for intergeneric conjugation to *S. coelicolor* strains [63].

All *E. coli* strains were grown in Luria-Bertani (LB) broth with the addition of antibiotics to maintain plasmid constructs as follows: chloramphenicol (25 µg/ml) for pBAD33, kanamycin (35 µg/ml) for pET28 and apramycin (50 µg/ml) for pGusT-E*. Bacteria carrying pBAD33 with DarT were grown in the presence of 0.8 % glucose to prevent toxin expression. All *E. coli* strains were grown at 37 °C (unless otherwise indicated).

*S. coelicolor* strains were grown in a liquid Complete regeneration medium (CRM) [64] and on solid Mannitol soya flour (MS) medium [65]. All *S. coelicolor* strains were grown at 30 °C.

### 4.3 Protein expression and purification

TaqDarT was expressed in BL21 cells grown in LB media; protein expression was induced with 0.8 % arabinose for 1.5 hr at 37 °C. LB medium was supplemented with glucose before induction to inhibit basal expression. DarT proteins were purified using TALON affinity resin (Clontech). A more detailed explanation of DarT and DarG expression and purification is given in [35]. SCO6735 was expressed and purified as previously described in [17]. The same purification protocol was used for SCO6735 mutants. Protein concentrations were determined using molar extinction coefficients and 280 nm absorption as measured by NanoDrop (DeNovix). The purity of SCO6735, DarG and TARG1 can be seen in Supplementary Fig. 8.

### 4.4 Isothermal CD experiments

Spectra were collected using parameters: cuvette 0.02 cm, selectivity standard, data pitch 0.2 nm, band width 1 nm, response 1 *sec*, measurement range 190–260 nm, 3 accumulations, scanning speed 50 nm/min, temp. = 30 °C. Each spectrum was corrected by subtraction of the baseline. The spectra are represented as MRE (Molar residue ellipticity) values to normalise the effect of concentration differences. Final average spectra were smoothed using the adjacent averaging method (origin7.5.) in 50 points. The HT voltage applied to the detector for all the collected data was lower than 600 V. Average CD isothermal spectra were calculated for samples in 50 mM phosphate buffer, pH 7.4, 100 mM ammonium sulphate, from two or multiple replicas. CD measurements were performed on CD Spectrometer Jasco J-815.

### 4.5 Toxicity assays

A toxicity assay in *E. coli* was performed as previously described [35]. For the assay in *S. coelicolor*, genes encoding DarT and DarT E160A with the addition of 5′-His tag coding sequence were re-cloned into pGusT-E* and integrated into the chromosome of *S. coelicolor* WT and *S. coelicolor* Δ6735 strains. Using theophylline-dependent riboswitches this system enables conditional gene expression in *S. coelicolor*. For this purpose, we had to redesign our previously constructed *S. coelicolor* Δ6735 strain [17], since the disruption cassette used for gene inactivation by the PCR-targeting system carries an apramycin resistance gene which interferes with further procedure. FLP recombinase-mediated excision [66], [67] was used for the removal of the central part of the disruption cassette leaving just an in-frame scar sequence with no antibiotic resistance. For the expression of DarT and DarT E160A in *S. coelicolor* WT and *S. coelicolor* Δ6735, the strains were streaked out on MS agar plates supplemented with 4 mM theophylline. Growth was monitored for 96 h.

### 4.6 Phylogenetic analysis

Macrodomains of selected protein sequences were aligned using the MUSCLE algorithm [68] and their phylogenetic relationship was analysed using MEGA X [69] with the Maximum Likelihood method and Whelan and Goldman model [70]. The tree is drawn to scale, with branch lengths measured in the number of substitutions per site. The numbers associated with branches are bootstrap values based on 1000 bootstrapping replications (values higher than 50 % are shown at the branching points). The species' full names and accession numbers of the 38 macrodomain sequences involved are listed in Supplementary Table 1.

### 4.7 Protein extraction and western blot analysis

*S. coelicolor* strains spores were inoculated in 1 ml of CRM and after 24 h transferred into 5 ml of fresh medium. To synchronise the growth, all strains were grown without apramycin. After 24 h of growth, biomass was transferred into 50 ml of fresh medium and continued to grow for the next 24 h when 4 mM theophylline was added. 24 h after induction, the biomass was collected by centrifugation and used for the extraction of total cellular proteins. The traces of CRM medium were rinsed out with washing buffer (25 mM Tris-HCl (pH 7.5) and 500 mM NaCl) and the biomass was re-suspended in the same buffer with the addition of 10 mM imidazole and 1 mg/ml lysozyme. After sonication, cell lysate was centrifuged at 13 000 g for 30 min at 4 °C. For the enrichment of His-tagged proteins, we used TALON affinity resin (TaKaRa) and a gravity-flow-based protocol. After the cell lysate passed through the column, the column was rinsed with washing buffer with the addition of 10 mM imidazole and the bound proteins were eluted with elution buffer (washing buffer with 200 mM imidazole). The eluates were desalted and concentrated using Amicon Ultra Centrifugal Filter Units (<10 K). For the western blot analysis, concentrated His-tagged proteins were separated by SDS-PAGE and stained with CBB. Identical samples, run in parallel, were transferred to the PVDF membrane (Amersham) and the proteins were visualised using an anti-His antibody (TaKaRa) and ECL detection system (Amersham).

### 4.8 Oligonucleotide ADP-ribosylation and de-ADP-ribosylation assays

Oligonucleotides were synthesized by Metabion. The sequences of substrate oligonucleotides ON-dT (TCTC): GAGCTGTACAAGTCAGATCTCGAGCTC and polyT-G (TT-G-TT): TTTTTTTTGTTTTTTTTT. ADP-ribosylation reactions were performed in 50 mM Tris-HCl pH 8, and 50 mM NaCl buffer at 30 °C for 45 min, unless stated otherwise. For the reactions, 0.3 µM DarT and 1 µM oligonucleotides in the presence of excess NAD^+^ (usually 3 mM) were used. The transferase reactions were stopped by denaturing at 95 °C for 5 min, cooled on ice, and the mixture containing hydrolases (DarG, TARG1 or SCO6735) was added and incubated at 30 °C for 30 min. Reactions were stopped by the addition of loading dye and denaturing at 95 °C for 5 min. Samples were resolved on 15 % polyacrylamide 8 M urea gel, stained with SYBR Gold and visualised under UV light.

### 4.9 Protein ADP-ribosylation and de-ADP-ribosylation assays

PARP E988Q mutant (1 µM) was auto-ADP-ribosylated in the presence of ^32^P-NAD^+^ at 10 kBq/reaction, 15 µM NAD^+^, 5 µM PARP-activating DNA for 30 min at 37 °C in PARP-assay buffer (50 mM Tris-HCl, 200 mM NaCl, 5 mM MgCl_2_, 1 mM DTT). Reactions were stopped and immediately used for de-ADP-ribosylation reactions with 1 µM SCO6735 WT and its mutants (or TARG1, 1 µM, as positive control) for 30 min at 30 °C. Reactions were stopped by denaturing at 95 °C, for 5 min. Samples were analysed by 15 % SDS-polyacrylamide gel electrophoresis followed by CBB staining and autoradiography.

### 4.10 Molecular dynamics simulations

Starting from the crystal structure of the SCO6735 apoprotein determined at 1.6 Å (PDB 5E3B) [17], the following systems were prepared for computational simulations: (i) apo structure of SCO6735 protein and its complexes with (ii) ADPr, (iii) cognate, (iv) non-cognate substrate. Systems (ii), (iii) and (iv) were generated as the results of the docking procedure described in the next section. Active site flexible loops that were missing in the X-ray structure were built using SWISS-MODEL [71]. Polar hydrogen atoms were added by H++ software version 3.2. [72], [73], [74], which adds protons to the input structure according to the calculated ionization states of its titratable groups at the user-specified pH, in our case pH was set to 7.5. Nonpolar hydrogen atoms were added with *tleap*, which is part of the Amber 16 program package [75]. Parameterization of protein atoms was accomplished with the amber ff14SB force field [76], while product and cognate and non-cognate substrates were parameterized in *xleap* using leaprc.ff14SB.redq force field. Protein was placed in the centre of the box (66–75, 68–73 and 62–70 Å) filled with TIP3P water molecules [77]. The system was neutralized by adding Na^+^ or Cl^−^ ions in *tleap*. The resulting systems consisted of ca. 25 000 atoms.

Geometric optimisation (energy minimisation) was carried out in four cycles. Each cycle consisted of 1000 steps of the steepest descent algorithm followed by 4000 steps of a conjugate gradient algorithm. In the first cycle, solvent molecules and ions were relaxed, while the protein atoms were constrained using a harmonic potential with a force constant of 100 kcal/(mol·Å^2^). In the second cycle, the same constraint was put on all non-hydrogen atoms of the protein; the goal of this cycle was the relaxation of hydrogen atoms. In the third cycle, the constraint was put only on the protein backbone atoms with the force constant of 100 kcal/(mol·Å^2^), respectively. In the fourth cycle, no constraints were applied.

After energy minimization, the systems were subjected to molecular dynamics (MD) simulations. During the first 300 ps of simulation of each system, protein atoms were constrained (32 kcal/(mol·Å^2^)) and the volume was kept constant, while the temperature was linearly increased from 0 K to 303 K. After the initial 300 ps, systems were simulated at constant pressure (101300 Pa) without any constraints on the atoms and the temperature was kept constant at 303 K. All simulations were 200 ns long. The time step of the simulation was 1 fs, structures were sampled every 1 ps, and periodic boundary conditions (PBC) were applied. The geometry optimization and the MD simulations were conducted using AMBER 16 software. The trajectories were analysed using cpptraj [78] from the Amber program package and VMD [79] and Chimera visualisation programs [80]. Electrostatic surface analysis was performed with the APBS-PDB2PQR software suite (https://server.poissonboltzmann.org/) [81].

### 4.11 Molecular docking

The main purpose of the molecular docking calculations was to obtain SCO6735 complexes with the product, cognate and non-cognate substrate that will be subjected to MD simulations. The crystal structure of SCO6735 apoprotein (PDB 5E3B) was used as a docking target. The active site of SCO6735 was determined by comparison with its structural homologues: DarG from *T. aquaticus* (PDB 5M3E) and human TARG1/Corf130 protein (PDB 4J5S) [17]. Since the active site flexible loops were missing in the crystal structure they were built by SWISS-MODEL [71], we firstly examined their dynamics and positioning by MD dynamic simulation. For the molecular docking of the ADPr, cognate and non-cognate substrate, we used apoprotein conformation with the most open active site obtained after 0.1 ns of MD simulation of apoprotein (Supplementary Fig. 2A and B). Results of molecular docking of ADPr in predicted active site are in agreement with our expectations and can be easily compared to DarG:ADPr and TARG1:ADPr complex (Fig. 1A).

ADPr, cognate and non-cognate substrates were built in Maestro (Supplementary Fig. 3C) and then prepared with LigPrep from Schrodinger (Schrodinger Release 2021–4: LigPrep, Schrodinger, LLC, New York, NY, 2021). The parametrisation of all ligands was carried out in xleap from the Amber program package. To get a better insight into DNA binding we added a terminal ribose and phosphate group to the dT/dG nucleotide. A docking study was carried out using Autodock Vina [82] and Autodock tools 1.5.6 [83]. For the docking of the cognate substrate the grid size was set at 24x28x26 XYZ points and centred at (8.693, 4.842, −5.221); for non-cognate 24x38x24 and centred at (7.552, 3.945, −2.955) and for the ADPr 24x26x30 and centred at (8.712, 3.28, −7.675). Grid spacing was set to 1 Å. For the Autodock Vina study, an extended PDB format, termed PDBQT, was used for coordinate files, which include atomic partial charges and atom types. Torsion angles were calculated to assign the flexible and non-bonded rotation of the product and substrate. Best docking scores were examined and used as starting structures for molecular dynamics simulations. Docking scores for ADPr, dT-ADPr and dG-ADPr were: −8.3 kcal/mol, −8.6 kcal/mol and −8.8 kcal/mol, respectively. Obtained complexes are shown in Supplementary Fig. 3D-F.

To predict DNA binding, several docking calculations of DNA molecules on the SCO6375 active site were conducted using the Haddock web server [54], [55]. DNA molecule from DarT:DNA crystal structure (PDB 7OMY) was used, and the most representative SCO6735:dT-ADPr structure obtained after 50 ns of MD simulation was used as the target protein structure.

## CRediT authorship contribution statement

**Andrea Hloušek-Kasun:** Conceptualization, Investigation, Formal analysis, Writing – original draft. **Petra Mikolčević:** Conceptualization, Investigation, Formal analysis, Writing – original draft, Funding acquisition. **Johannes Gregor Matthias Rack:** Conceptualization, Writing – review & editing. **Callum Tromans-Coia:** Investigation. **Marion Schuller:** Writing – review & editing. **Gytis Jankevicius:** Investigation. **Marija Matković:** Data curation. **Branimir Bertoša:** Writing – review & editing, Validation. **Ivan Ahel:** Writing – review & editing, Funding acquisition. **Andreja Mikoč:** Conceptualization, Writing – review & editing, Funding acquisition.

## Declaration of Competing Interest

The authors declare that they have no known competing financial interests or personal relationships that could have appeared to influence the work reported in this paper.
