# Supplementary material for: Streptomyces coelicolor macrodomain hydrolase SCO6735 cleaves thymidine-linked ADP-ribosylation of DNA
Source: Comput Struct Biotechnol J. 2022 Aug 8;20:4337–50. doi: 10.1016/j.csbj.2022.08.002 (PMC9411070; doi:10.1016/j.csbj.2022.08.002)
Supplement: Supplementary data 1 [file mmc1.docx]

# Supplementary Information


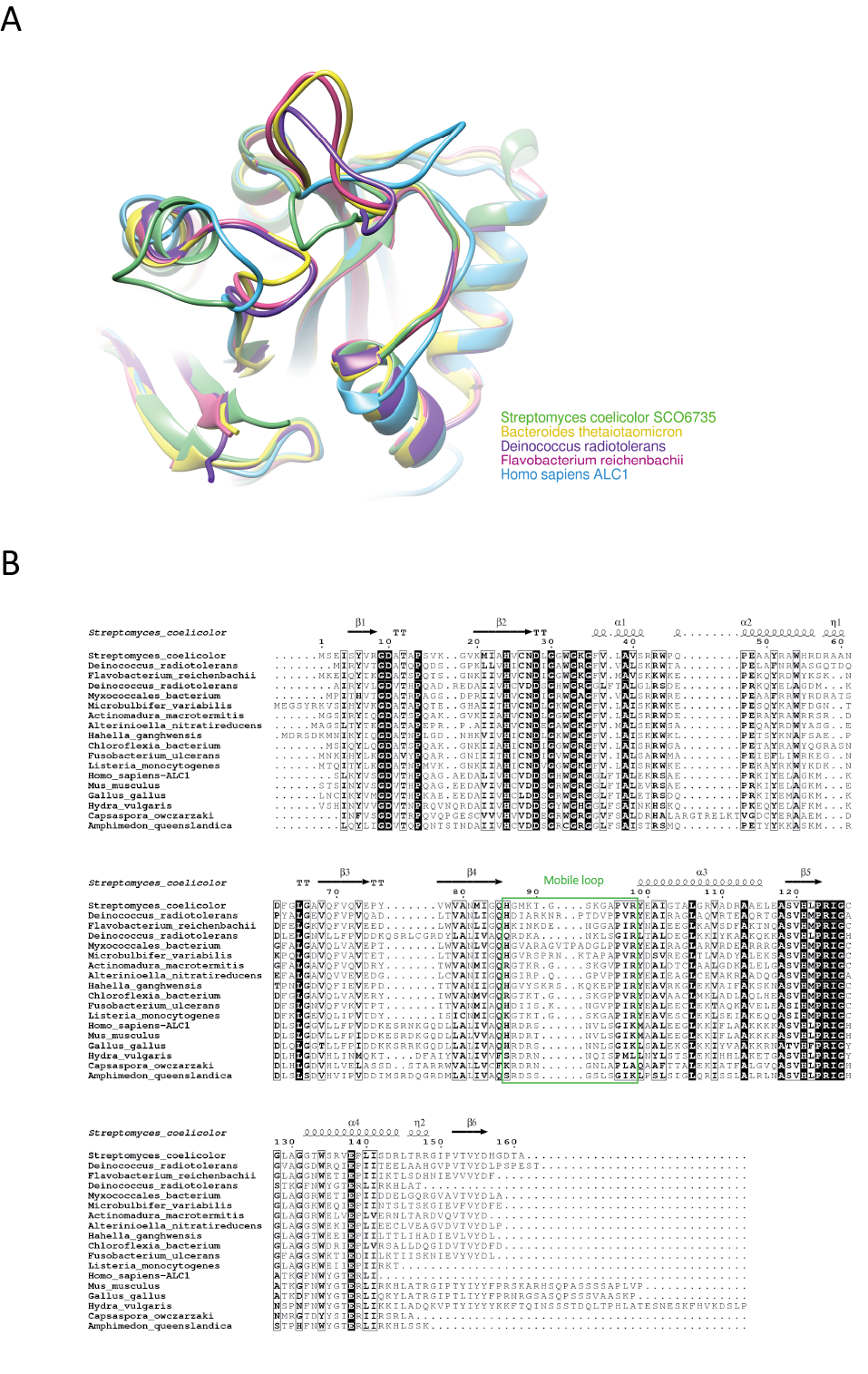


**Supplementary Figure 1. The longer mobile loop is characteristic of macrodomain proteins within the ALC1-like class.** (A) Superimposition of predicted 3D structures of macrodomain proteins within the ALC1 class presented in the phylogenetic tree in Fig 1C. Structure prediction was carried out using the Alpha fold [[84](#_ENREF_84)]. (B) More extensive sequence alignment of macrodomain proteins from the ALC1 class. Amino acids that are within the mobile loop are boxed with a green rectangle.

## SCO6735 specificity continued (Supplementary Figure 2)

Method: For testing the residue specificity of SCO6735, model substrates and transferases were used (FKBP1 substrate with ARTC2.2 transferase for Arg, auto-modified PARP1 WT for Ser and PARP1 EQ for Glu/Asp) in the same way as described in Protein ADP-ribosylation and de-ADP-ribosylation assays section. After transferase reaction, samples were immediately assayed (without denaturing) with SCO6735 or the control hydrolases (hARH1 (Arg), hARH3 (Ser), hTARG1 (Glu/Asp)) which were all present at the concentration of 1 µM.


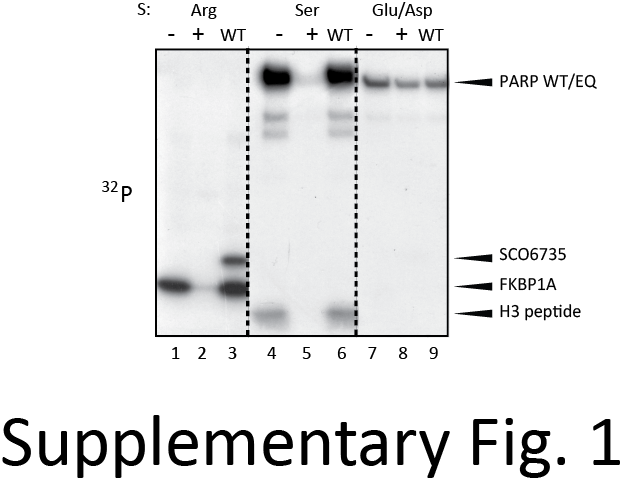


**Supplementary Figure 2. SCO6735 specificity continued.** SCO6735 WT activity on different protein substrates. From left to right: ADPr-Arg (FKBP1A (and SCO6735) modified by ARTC2.2 transferase), ADPr-Ser (H3 peptide and PARP1 modified by PARP1) and ADPr-Asp/Glu (PARP1 E988Q self-modified). Positive (+) control hydrolases were: hARH1 (Arg), hARH3 (Ser), hTARG1 (Glu/Asp)). The reactions were analysed by PAGE and autoradiography.


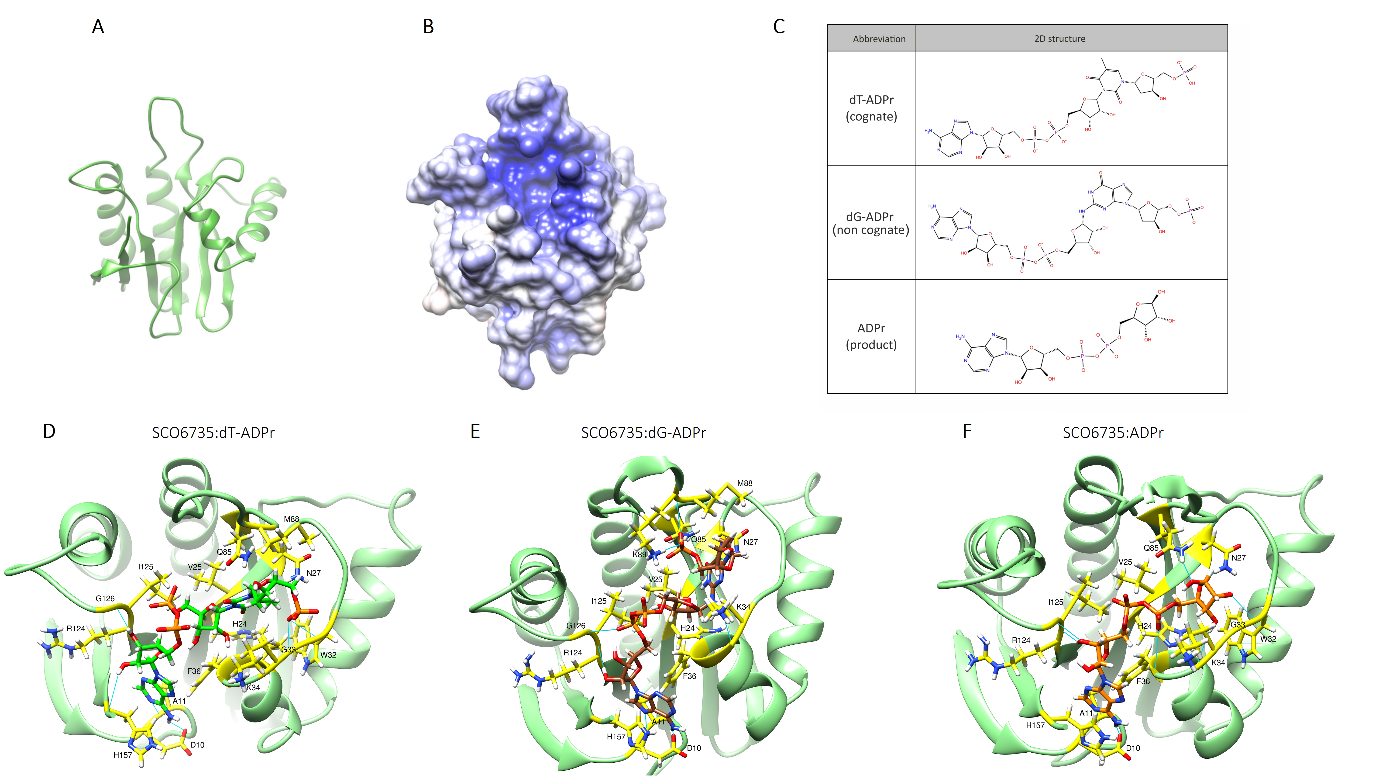


**Supplementary Figure 3. Conformations of SCO6735 in complex with its product/substrates after molecular docking.** (A, B) SCO6735 protein conformations used for molecular docking. (C) 2D structures of the product (ADPr), cognate (dT-ADPr) and non-cognate (dG-ADPr) substrate. (D) SCO6735:dT-ADPr, (E) SCO6735:dG-ADPr and (F) SCO6735:ADPr complex obtained after docking calculations and energy minimisation.


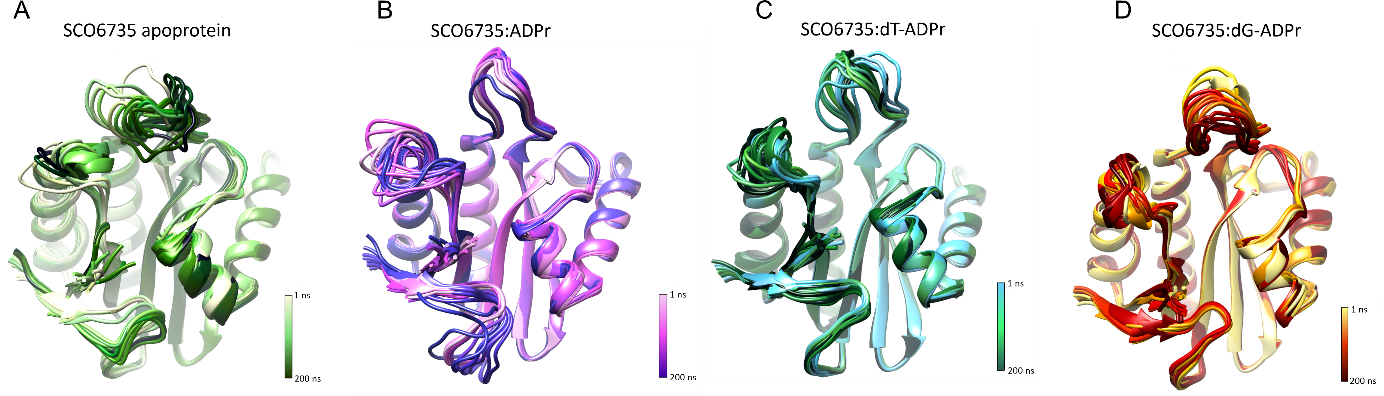


**Supplementary Figure 4. Dynamics of SCO6735 protein and its complexes with the product, cognate, and non-cognate substrates.** (A) Superimposition of twenty average structures of SCO6735 apoprotein, complex with the ADPr (B), cognate (C) and non-cognate substrate (D). Average structures were calculated every 10 ns over 200 ns of MD simulation. For the clarity of the figure, average structures of ligands were omitted.


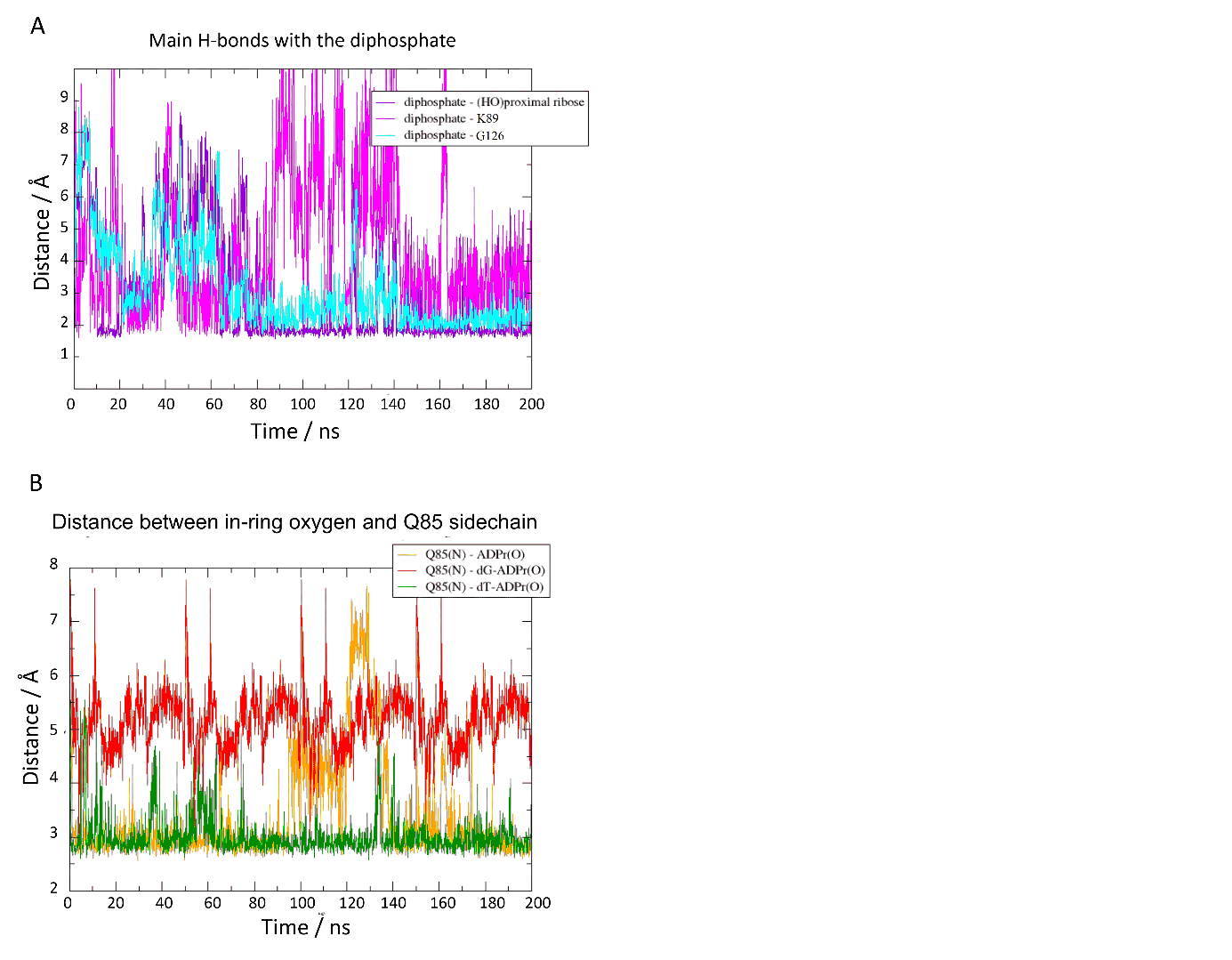


**Supplementary Figure 5. Distances between key atoms in the SCO6735 active site.** (A) Distance between oxygen in the diphosphate and hydrogen atoms in Lys89 and Gly126 that make stable H-bonds. The most stable interaction is the intermolecular H-bond between the diphosphate and the distal ribose (purple). (B) Distance between in-ring oxygen atom in distal ribose and Gln85 sidechain amino group in all three simulated complexes, SCO6735:ADPr (orange), SCO6735:dT-ADPr (green) and SCO6735:dG-ADPr (red). Stable distance (within a range of an H-bond) is the most pronounced in the case of the complex with the cognate substrate.


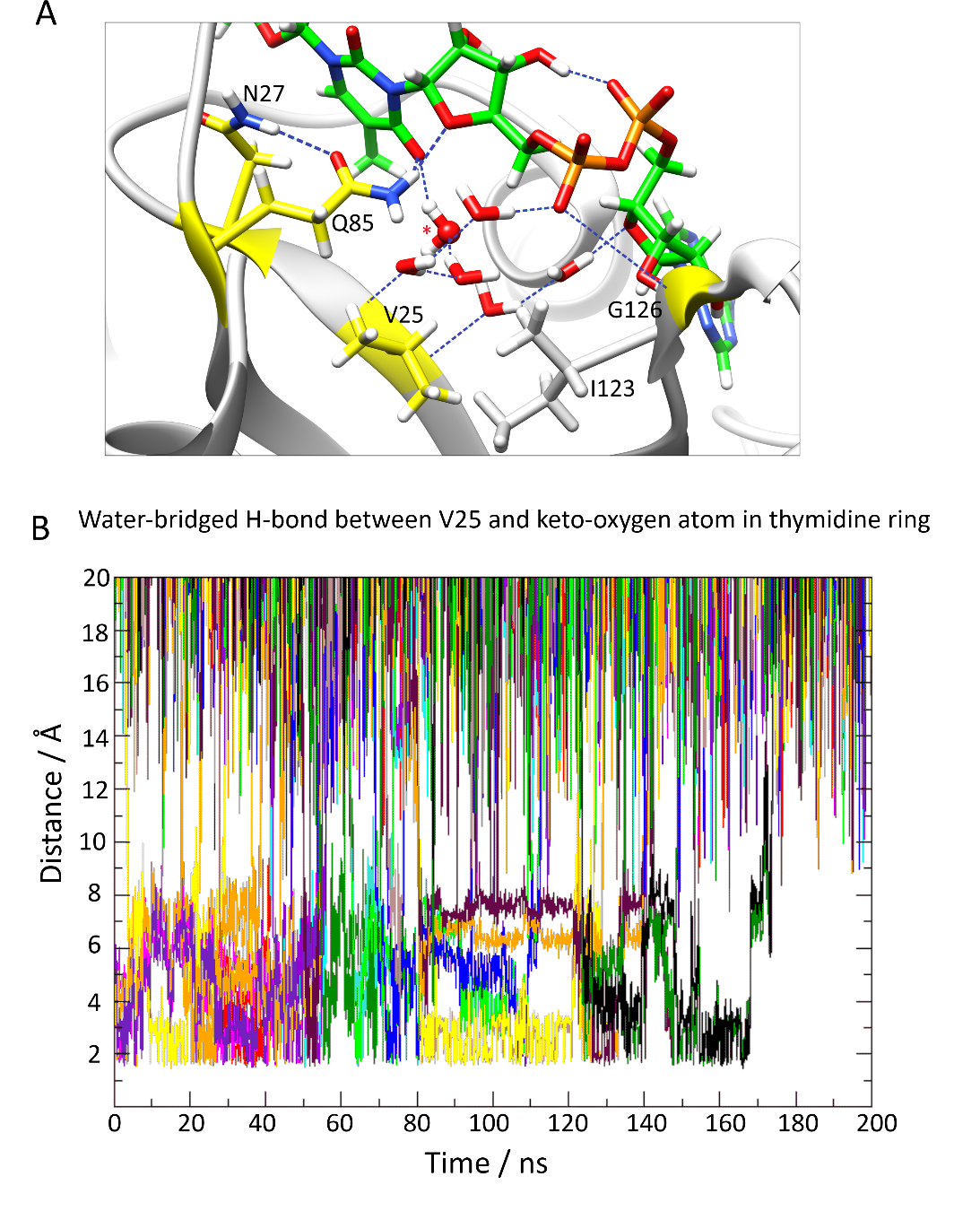


**Supplementary Figure 6. Water-molecule network in the SCO6735 active site.** (A) Detailed analysis of the active site water-molecule network positioned between the diphosphate, Gln85 and Val25. This network of water molecules includes a water molecule snugged in between Val25 and the keto-oxygen in the thymidine ring during almost all MD simulations. The water molecule that could be involved in catalysis is depicted by sticks and ball representation and marked with the red asterisk. (B) Distance calculations between Val25, the keto-oxygen atom in the thymidine ring and water molecules that interchange between them during all MD simulations.


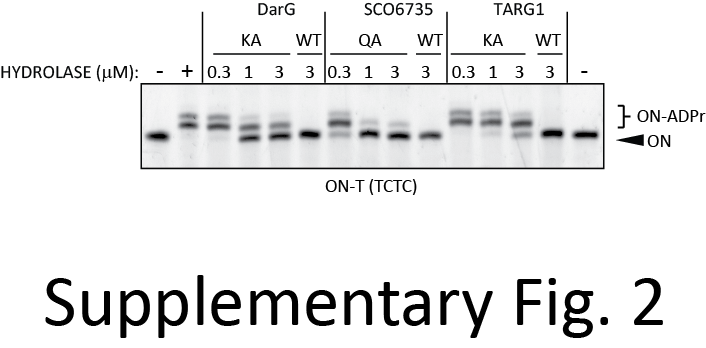


**Supplementary Figure 7. Interaction-inhibition of DarT.** SCO6735, DarG and TARG1 proteins or their inactive mutants (Q85A, K80A and K84A, respectively) were added together with DarT (0.3 µM) and the TCTC oligo (0.5 µM). Reactions were incubated at 30°C for 1 hr and analysed by the gel-shift assay on polyacrylamide urea gel. The negative control is TCTC non-modified oligo alone and the positive one is with the addition of DarT and no hydrolase.


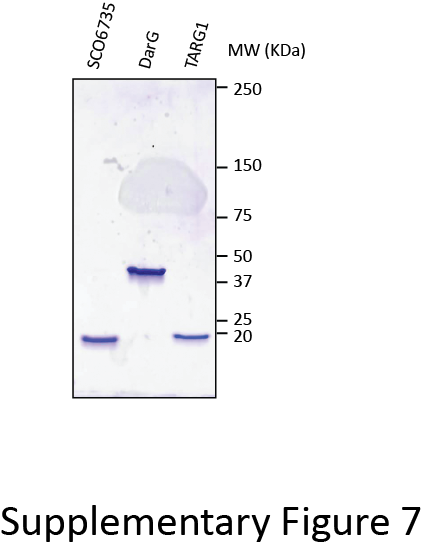


**Supplementary Figure 8. Purified SCO6735, DarG and TARG1 on SDS-PAGE.** Recombinant SCO6735, DarG and TARG1 were purified (as described in Materials and Methods) and subjected to SDS-PAGE, stained with CBB (25 µM each).


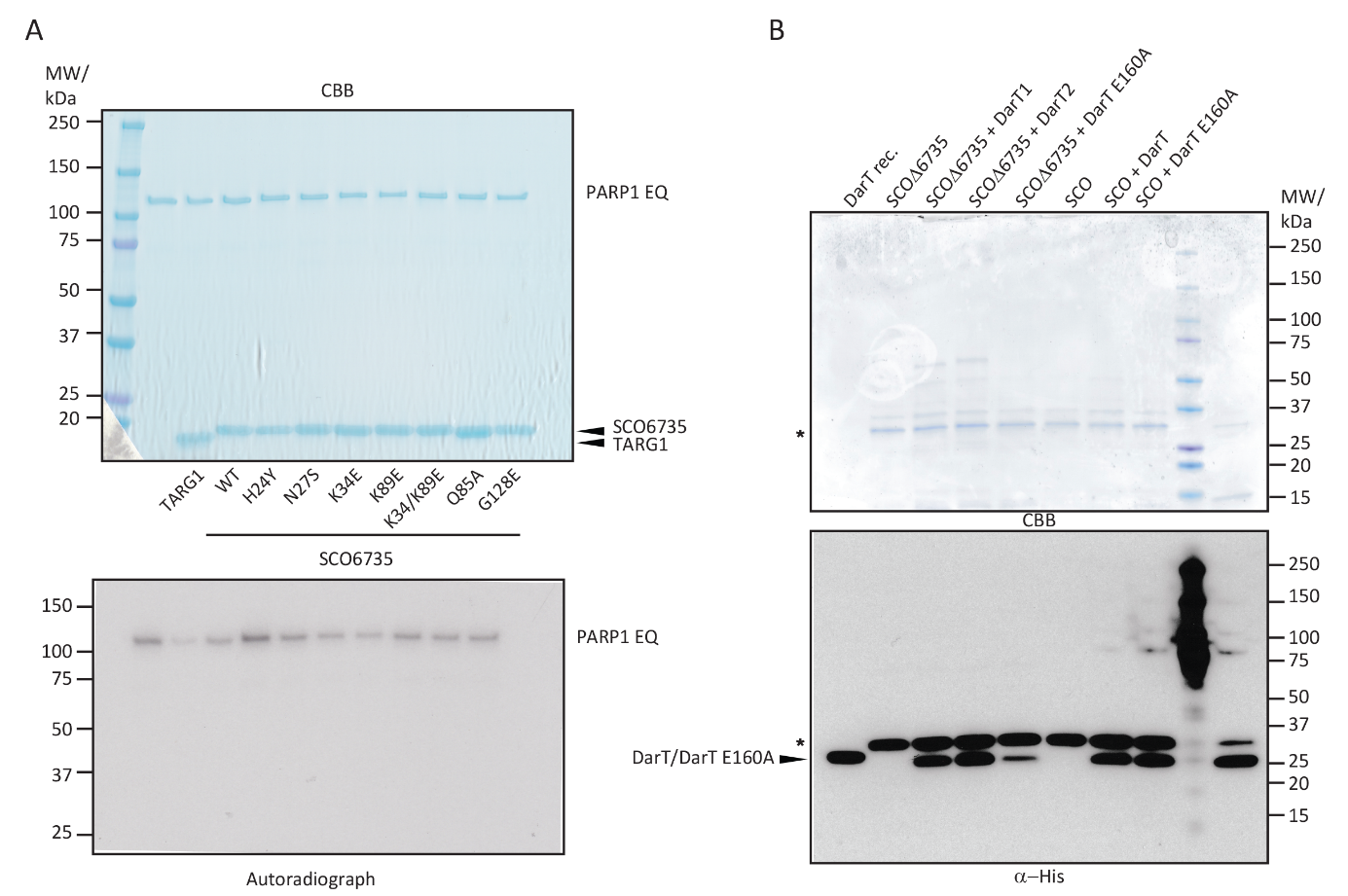


**Supplementary Figure 9. CBB staining, autoradiograph, and western blot for Figures 4 and 5.** (A) Uncropped versions of the images shown in Fig. 4B; CBB stained SDS-PAGE gel with PARP1 EQ, TARG1 and SCO6735 (WT and mutants) proteins and autoradiograph showing the same gel exposed to the X-ray film. (B) Uncropped versions of the images shown in Fig. 5B; CBB stained SDS-PAGE gel with His-tagged enriched total cellular proteins from indicated *S. coelicolor* strains and western blot with anti-His antibody below. Asterisk marks the background band seen in all samples.

**Supplementary Figure 10. Conservation of SCO6735 key catalytic residues in SCO6735, ALC1, TARG1 and DarG homologues.** Macrodomain sequence alignment includes representatives of several bacterial phyla: Actinobacteria (*Actinomadura macrotermitis* (MQY05003), Bacteroidetes (*Bacteroides thetaiotaomicron* (KAB4474009) and *Flavobacterium reichenbachii* (WP_035681448)), Chloroflexi (*Chloroflexia bacterium* (KAB8140059)), Cyanobacteria (*Cyanosarcina radialis* (MBW4619270)), Deinococcus/ Thermus (*Deinococcus radiotolerans* (WP_189068660)), Firmicutes (*Acetivibrio mesophilus* (WP_128706582) and *Listeria monocytogenes* (EAE7966195)) and Proteobacteria (*Alterinioella nitratireducens* (WP_172320777), *Neisseriaceae bacterium* (MCC5775837), *Hahella ganghwensis* (WP_211210729), *Oleibacter marinus* (WP_076517241), *Marinobacter xestospongiae* (WP_248166955), and *Myxococcales bacterium* (RYE90143)), cnidarian *Nematostella vectensis* with three homologues (listed in order of appearance: EDO27931, XP_032227118, XP_032219826) and other species listed in Supplementary Table 1. Valine (or isoleucine) and glutamine important for the catalytic mechanism and SCO6735 activity are conserved among SCO6735 and SCO6735/ALC1 homologues, respectively (boxed in red).


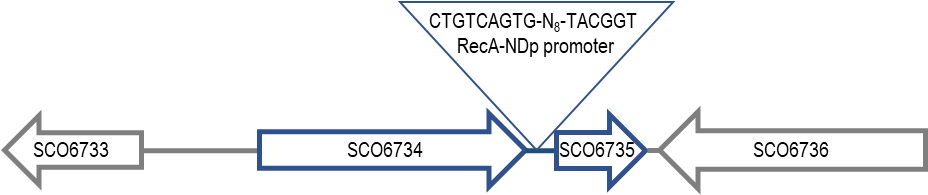


**Supplementary Figure 11. SCO6735 gene map.** SCO6734 and SCO6735 genes are in the same orientation and potentially could be in the same operon. SCO6735 gene is preceded by the conserved RecA-NDp promoter.


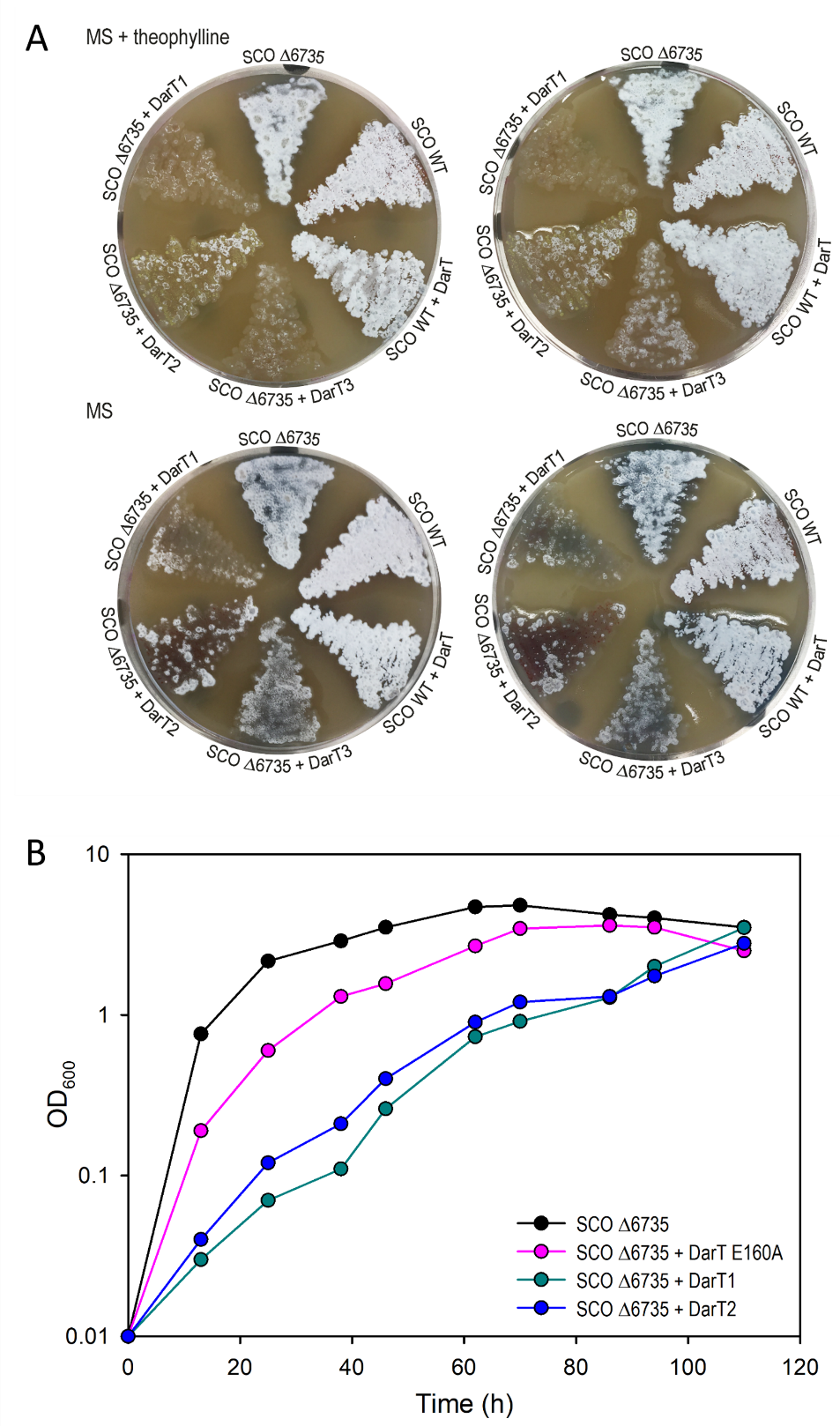


**Supplementary Figure 12. *S. coelicolor* strains expressing DarT and DarT E160A grown in liquid culture.** (A) *S. coelicolor* WT and SCO6735 deficient strain (SCOΔ6735) expressing DarT (DarT1-3) grown on MS plates after induction in liquid culture. The strains were grown in liquid CRM 24 h after theophylline induction and then spread in duplicates on MS agar plates with or without 4 mM theophylline. Growth on the plates was monitored for 48 h. (B) Growth curves of SCOΔ6735 strains expressing DarT and DarT E160A. The strains were grown for 110 hours in liquid CRM supplemented with 4 mM theophylline. Aliquots were taken at the indicated time intervals and the optical density at 600 nm was determined. The growth retardation effect was observed for the strains expressing DarT (DarT1 and DarT2). Although weaker, the effect was also observed for the strain expressing catalytic mutant DarT E160A corroborating that this mutant is not completely inactive and possesses weak ADP-ribosylation activity [31].

Supplementary Table 1. NCBI accession numbers of proteins used for the phylogenetic tree (Fig. 1B).

| Macrodomain family/Species | NCBI accession |
| --- | --- |
| **ALC1-like** | |
| *Homo sapiens* ALC1 | AAO49505 |
| *Streptomyces coelicolor* SCO6735 | CAB40685 |
| *Bacteroides thetaiotaomicron* | KAB4474009 |
| *Deinococcus radiotolerans* | WP_189068660 |
| *Flavobacterium reichenbachii* | WP_035681448 |
| **TARG1** | |
| *Homo sapiens* TARG1 | NP_001316613 |
| *Amphimedon queenslandica* | XP_019851965 |
| *Fusobacterium mortiferum* | AVQ19530 |
| *Magnetococcales bacterium* | MBA42542 |
| *Paenibacillus durus* | WP_042208697 |
| **DarG** | |
| *Escherichia coli* | CAS08640 |
| *Deinococcus indicus* | WP_088249216 |
| *Mycobacterium tuberculosis* | WP_193692490 |
| *Streptomyces scopuliridis* | WP_030355665 |
| *Thermus aquaticus* | WP_003046167 |
| **Macro2** | |
| *Amphimedon queenslandica* | XP_003388308 |
| *Physcomitrium patens* | XP_024388278 |
| *Saccharomyces cerevisiae* | AJS61953 |
| *Deinococcus radiotolerans* | WP_189069767 |
| *Herpetosiphon aurantiacus* | ABX02660 |
| *Streptomyces griseus* | WP_030758248 |
| **MacroD** | |
| *Homo sapiens* MACROD1 | AAH03188 |
| *Homo sapiens* MACROD2 | A1Z1Q3 |
| *Amphimedon queenslandica* | XP_019851841 |
| *Escherichia coli* YmdB | CAD6017033 |
| *Deinococcus radiodurans* | WP_010888916 |
| *Oceanobacillus iheyensis* | WP_011066681 |
| *Streptomyces coelicolor* SCO6450 | CAA22759 |
| **MacroH2A** | |
| *Homo sapiens* H2A.1 | NP_613258 |
| *Homo sapiens* H2A.2 | NP_061119 |
| *Amphimedon queenslandica* | XP_003389250 |
| *Capsaspora owczarzaki* | XP_004347529 |
| **PARG** | |
| *Corallococcus macrosporus* | 013938184 |
| *Deinococcus radiodurans* | AAF12648 |
| *Flavobacterium reichenbachii* | WP_035681451 |
| *Herpetosiphon aurantiacus* | ABX04261 |
| *Streptomyces coelicolor* SCO0909 | CAB62700 |
| *Thermomonospora curvata* | ACY97295 |
